# Supplementary material for: Understanding the spread of agriculture in the Western Mediterranean (6th-3rd millennia BC) with Machine Learning tools
Source: Nat Commun. 2025 Jan 15;16:678. doi: 10.1038/s41467-024-55541-y (PMC11732979; doi:10.1038/s41467-024-55541-y)
Supplement: Supplementary file 1 — Supplementary Information [file 41467_2024_55541_MOESM1_ESM.pdf]

Supplementary information

Understanding the spread of agriculture in the Western Mediterranean (6th-3rd millennia BC) with Machine Learning tools

Castiello, Maria Elena* [1,2] MariaElena.Castiello@unil.ch

Russo, Emmanuele[3]

Martínez-Grau, Héctor[4]

Jesus, Ana[4]

Prats, Georgina[4, 5]

Antolín, Ferran[2,4]

[1] Institut d'archéologie et des sciences de l'antiquité, University of Lausanne, Switzerland.

[2] Division of Natural Sciences, German Archaeological Institute, Berlin, Germany.

[3] Institute for Atmospheric and Climate Science, ETH Zurich, Switzerland.

[4] Integrative Prehistory and Archaeological Science, Department of Environmental Sciences, University of Basel, Switzerland.

[5] ARQHISTEC, Grup d'Investigació Prehistòrica (GIP-UdL), Departament de Geografia, Història i Història de l'Art, Universitat de Lleida, Spain.

* Corresponding author

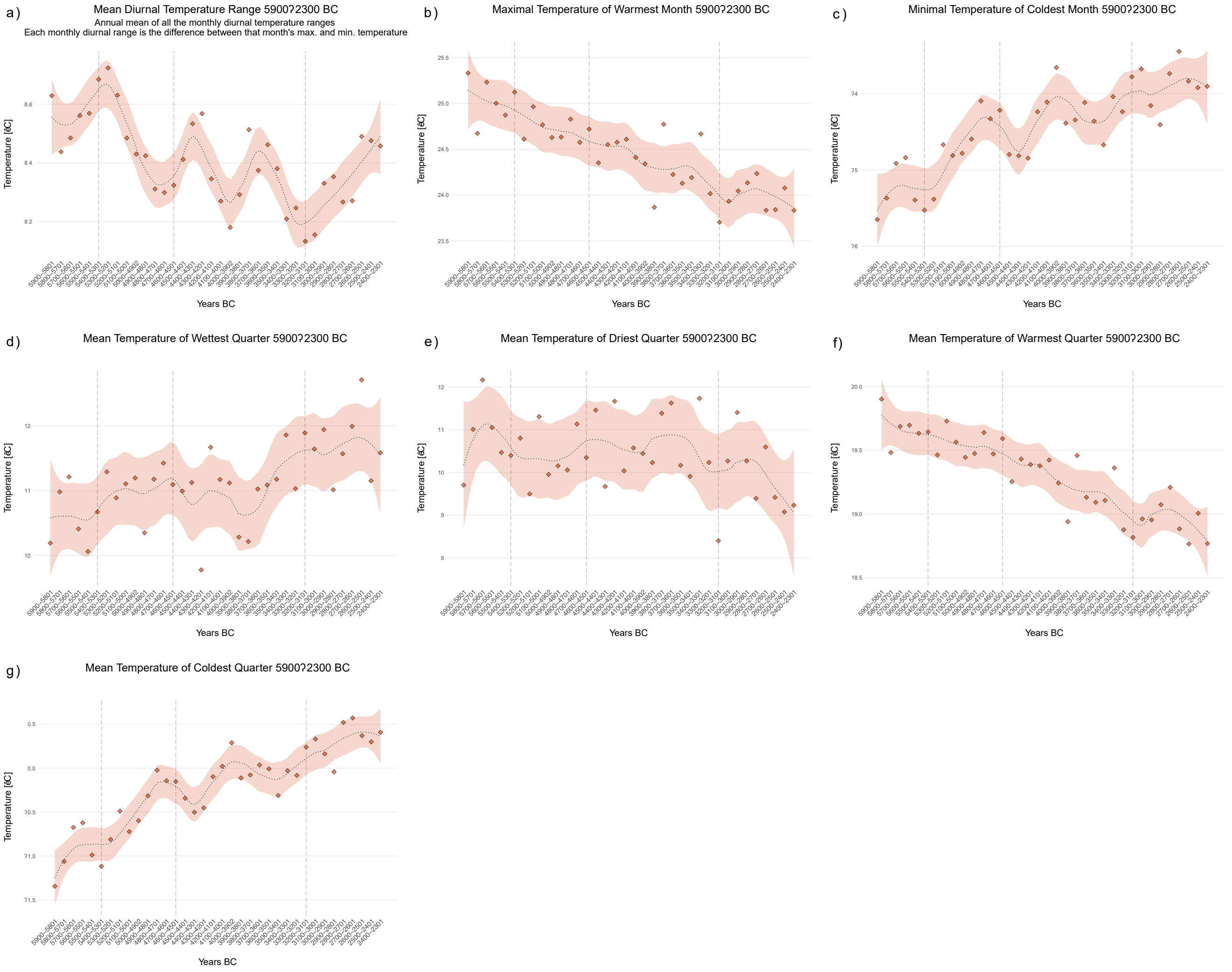

Supplementary Figure 1: Paleoclimate (Temperature related) variables derived from the *Chelsa-Trace 21k* dataset (Karger et al. 2020). From the upper left to the lower right panel: a) Mean Diurnal Temperature Range (BIO 02); b) Maximal Temperature of Warmest Month (BIO 05); c) Minimal Temperature of Coldest Month (BIO 06); d) Mean Temperature of Wettest Quarter (BIO 08); e) Mean Temperature of Driest Quarter (BIO 09); f) Mean Temperature of Warmest Quarter (BIO 10); g) Mean Temperature of Coldest Quarter (BIO 11). The graphs show the evolution of the BIO over the entire study area and period. The points represent the calculated mean value over the entire study area for each 100-year step. The dotted line represents the loess smooth with a span of 0.3 and the filled area the 95% confidence level interval (geom_smooth function from ggplot2 package in *R*). The vertical dashed lines indicate the limits of our 4 chrono-phases. Source data can be found on https://doi.org/10.5281/zenodo.14253277.

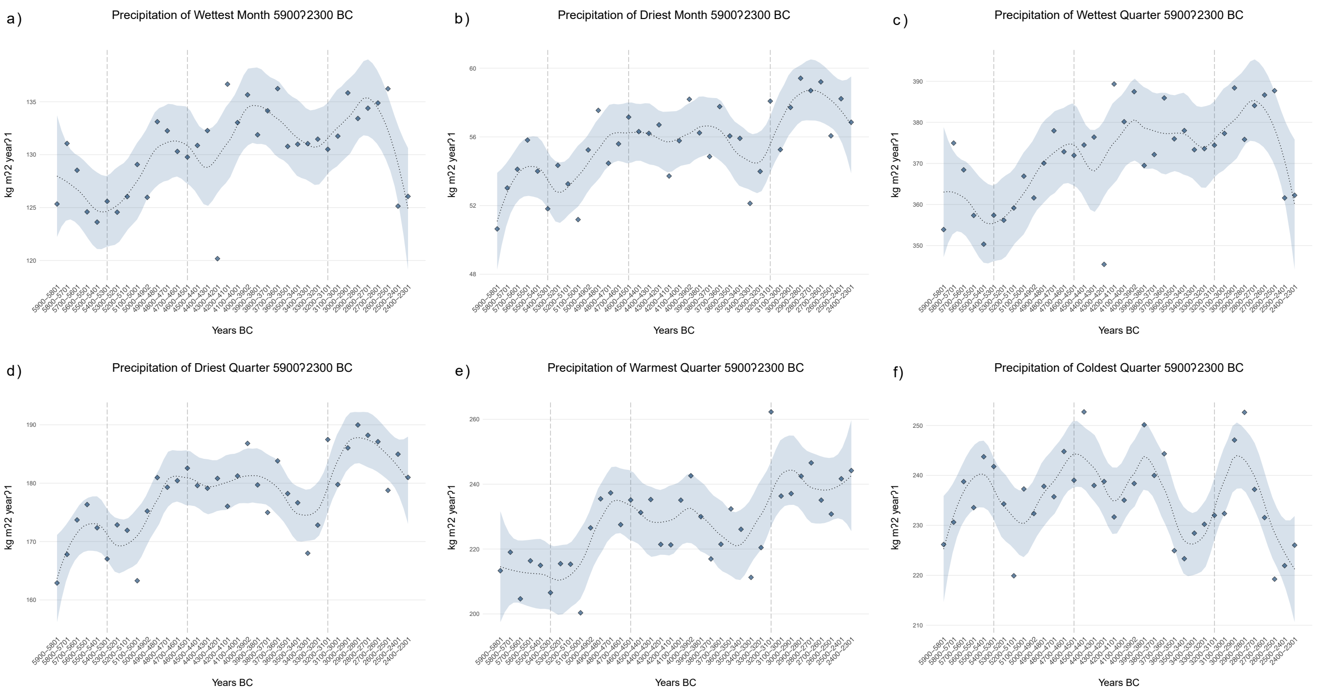

Supplementary Figure 2: Paleoclimate (Precipitation related) variables derived from the Chelsa-Trace 21k dataset (Karger et al. 2020). From the upper left to the lower right panel: a) Precipitation of Wettest Month (BIO 13); b) Precipitation of Driest Month (BIO 14); c) Precipitation of Wettest Quarter (BIO 16); d) Precipitation of Driest Quarter (BIO 17); e) Precipitation of Warmest Quarter (BIO 18); f) Precipitation of Coldest Quarter (BIO 19). The graphs show the evolution of the BIO over the entire study area and period. The points represent the calculated mean value over the entire study area for each 100- year step. The dotted line represents the loess smooth with a span of 0.3 and the filled area the 95% confidence level interval (geom_smooth function from ggplot2 package in R). The vertical dashed lines indicate the limits of our 4 chrono- phases. Source data can be found on https://doi.org/10.5281/zenodo.14253277.

Phase	Period
Phase 1	5900-5300 cal. BC
Phase 2	5299-4500 cal. BC
Phase 3	4499-3100 cal. BC
Phase 4	3099-2300 cal. BC

Supplementary Table 1: Chrono-Phases. The table shows the four phases with their chronological limits in which the archaeological and archaeobotanical dataset has been divided.

Supplementary Note 1

The division of the datasets in four Phases has been performed based on crop dynamics and as one of the tasks performed by the AgriChange research group, which research results and datasets are published in: Antolin et al. 2021, Jesus et al. 2021; Martinez-Grau et al. 2021; Antolin et al. 2024. To summarize the previous analyses performed: From each site with carpological information, the dominance of the species was semi-quantified in very abundant (5), abundant (3), present (1) and no data (0). This took into account the relative proportions of each crop at the site and its ubiquity, when quantified data was available. The crop dominance has been represented in the figure below, showing the polynomial trendlines of 3rd order of the various crops over time. Based on the trendlines of crop dynamics, four main time periods were defined: 5900-5300 cal BC, 5300-4500 cal BC, 4500-3100 cal BC and 3100-2300 cal BC.

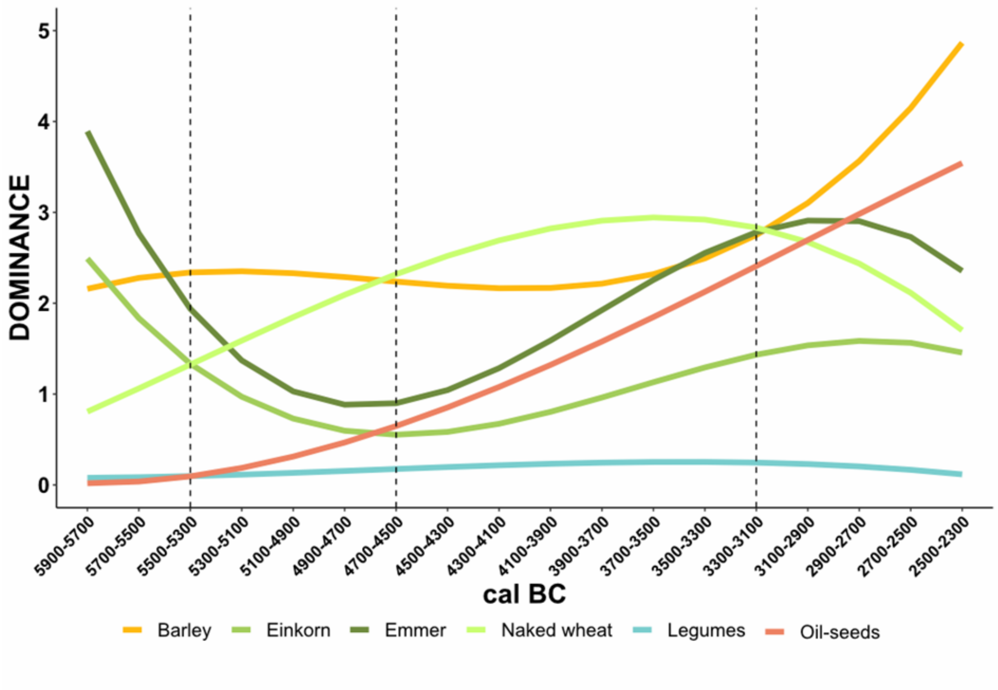

Site Type	Phase 01	Phase 02	Phase 03	Phase 04	Total	Percentage
Open Air	92	364	982	287	1725	50.5 %
Cave	81	251	330	135	797	23.3 %
Rock Shelter	102	215	157	47	521	15.3 %
Pile-Dwelling	6	53	87	90	236	6.9 %
Dolmen	1	2	58	36	97	2.8 %
Mine	0	3	33	4	40	1.2 %
Total	282	888	1647	599	3416	100 %

Supplementary Table 2: Site types and numbers. The table shows the total number and relative frequency in percentage of each site type.

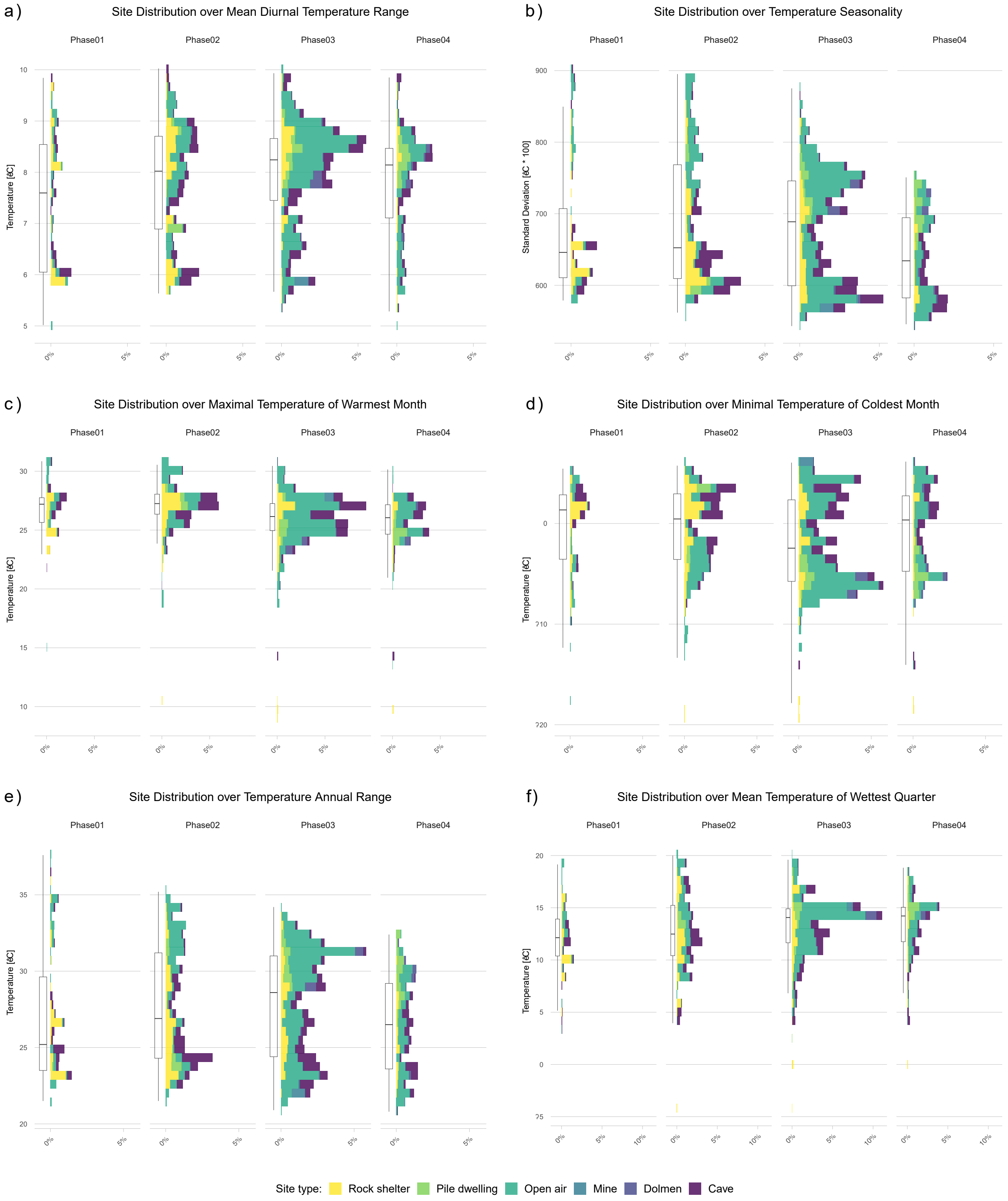

Supplementary Figure 3a: Distribution of Site types over temperature related paleoclimate variables per Phase. Each panel shows the overall distribution (box plot) of all sites and the distribution of site types (histograms) over Mean Diurnal Temperature Range (a), Temperature Seasonality (b), Maximal Temperature of Warmest Month (c), Minimal Temperature of Coldest Month (d), Temperature Annual Range (e), Mean Temperature of Wettest Quarter (f) per Phase. The histograms display 30 equally wide bins showing the percentual distribution of site types over the variables. The boxplots show medians, first and third quartiles (hinges), minimum and maximum values no further than 1.5*IQR from the hinge where IQR is the inter-quartile range (whiskers). Details about sample numbers are provided in Supplementary Table 2. Source data can be found on https://doi.org/10.5281/zenodo.14253277.

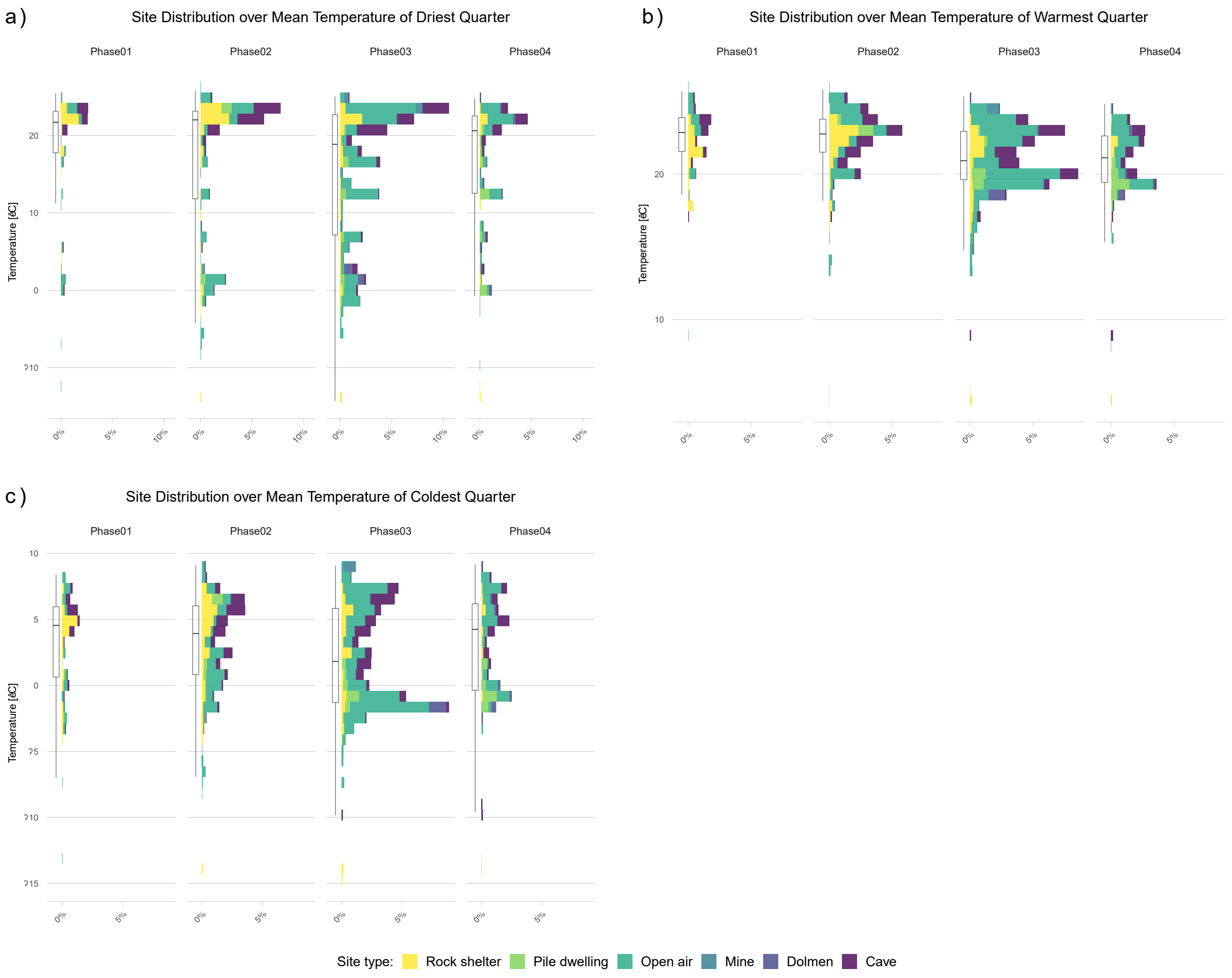

Supplementary Figure 3b: Distribution of Site types over temperature related paleoclimate variables per Phase. Each panel shows the overall distribution (box plot) of all sites and the distribution of site types (histograms) over Mean Temperature of Driest Quarter (a), Mean Temperature of Warmest Quarter (b), Mean Temperature of Coldest Quarter (c) per Phase. The histograms display 30 equally wide bins showing the percentual distribution of site types over the variables. The boxplots show medians, first and third quartiles (hinges), minimum and maximum values no further than 1.5*IQR from the hinge where IQR is the inter-quartile range (whiskers). Details about sample numbers are provided in Supplementary Table 2. Source data can be found on https://doi.org/10.5281/zenodo.14253277.

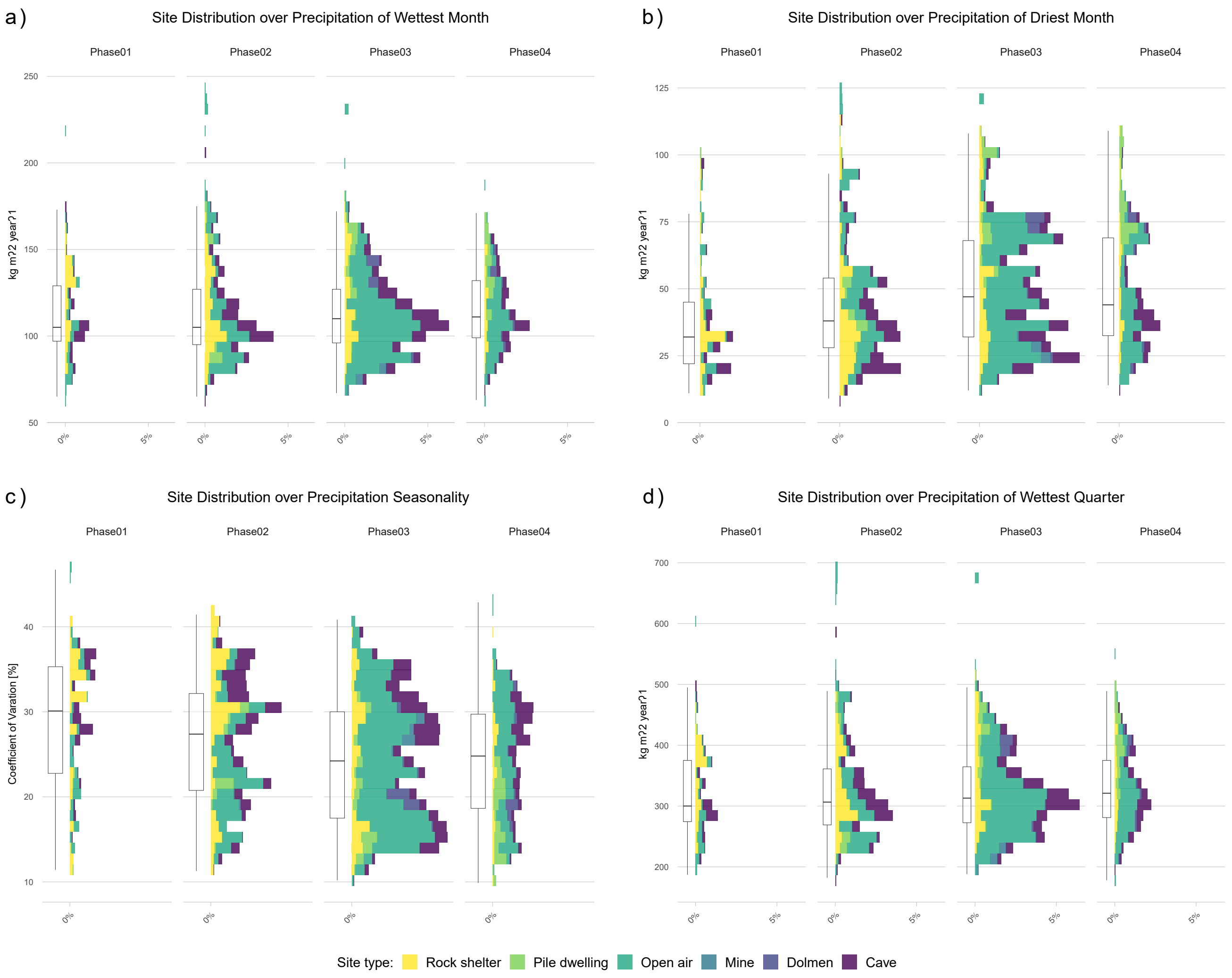

Supplementary Figure 3c: Distribution of Site types over precipitation related paleoclimate variables per Phase. Each panel shows the overall distribution (box plot) of all sites and the distribution of site types (histograms) over Precipitation of Wettest Month (a), Precipitation of Driest Month (b), Precipitation Seasonality (c), Precipitation of Wettest Quarter (d) per Phase. The histograms display 30 equally wide bins showing the percentual distribution of site types over the variables. The boxplots show medians, first and third quartiles (hinges), minimum and maximum values no further than 1.5*IQR from the hinge where IQR is the inter-quartile range (whiskers). Details about sample numbers are provided in Supplementary Table 2. Source data can be found on https://doi.org/10.5281/zenodo.14253277.

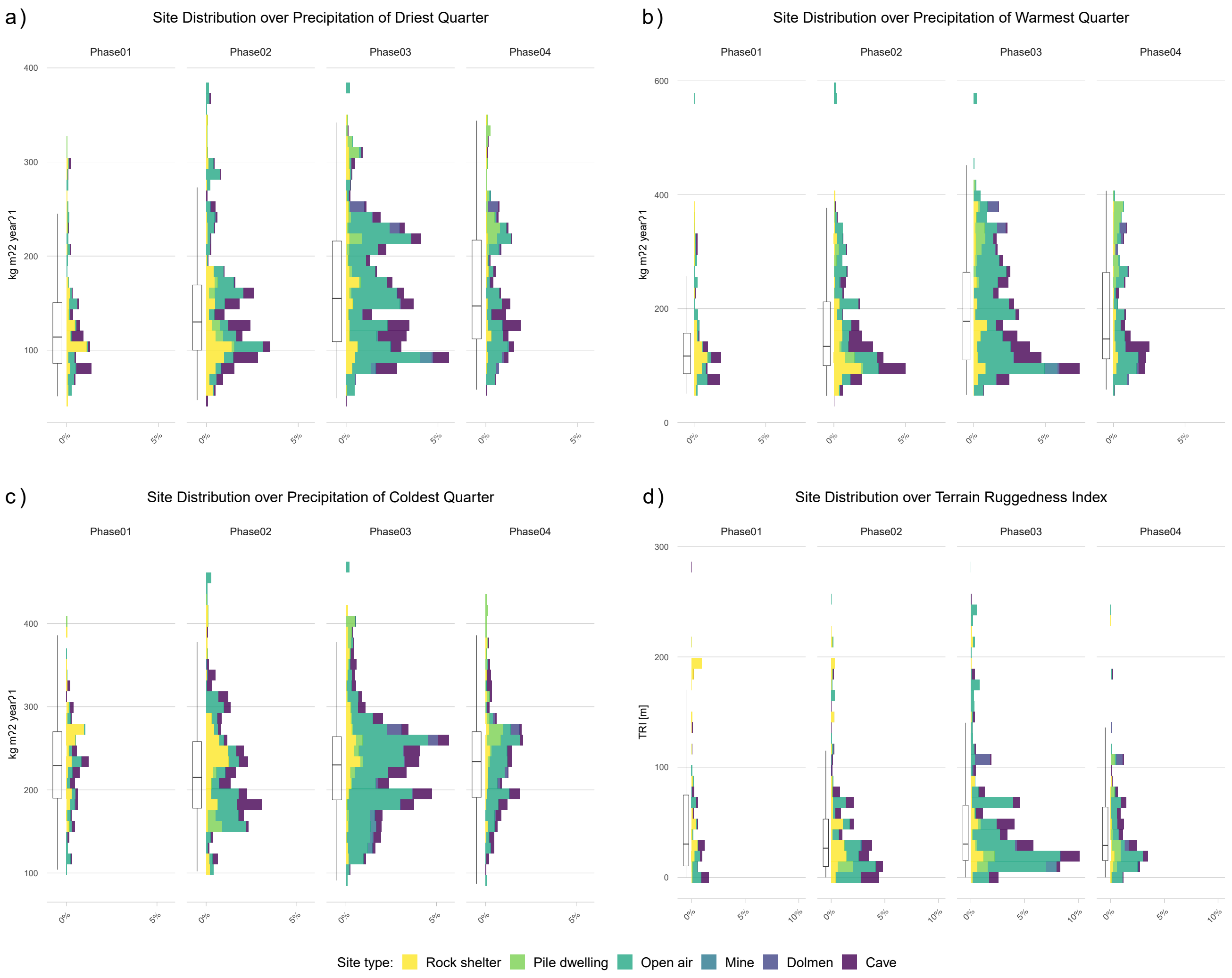

Supplementary Figure 3d: Distribution of Site types over precipitation related paleoclimate variables and Terrain Ruggedness Index per Phase. Each panel shows the overall distribution (box plot) of all sites and the distribution of site types (histograms) over Precipitation of Driest Quarter (a), Precipitation of Warmest Quarter (b), Precipitation of Coldest Quarter (c) and Terrain Ruggedness Index (d) per Phase. The histograms display 30 equally wide bins showing the percentual distribution of site types over the variables. The boxplots show medians, first and third quartiles (hinges), minimum and maximum values no further than 1.5*IQR from the hinge where IQR is the inter-quartile range (whiskers). Details about sample numbers are provided in Supplementary Table 2. Source data can be found on https://doi.org/10.5281/zenodo.14253277.

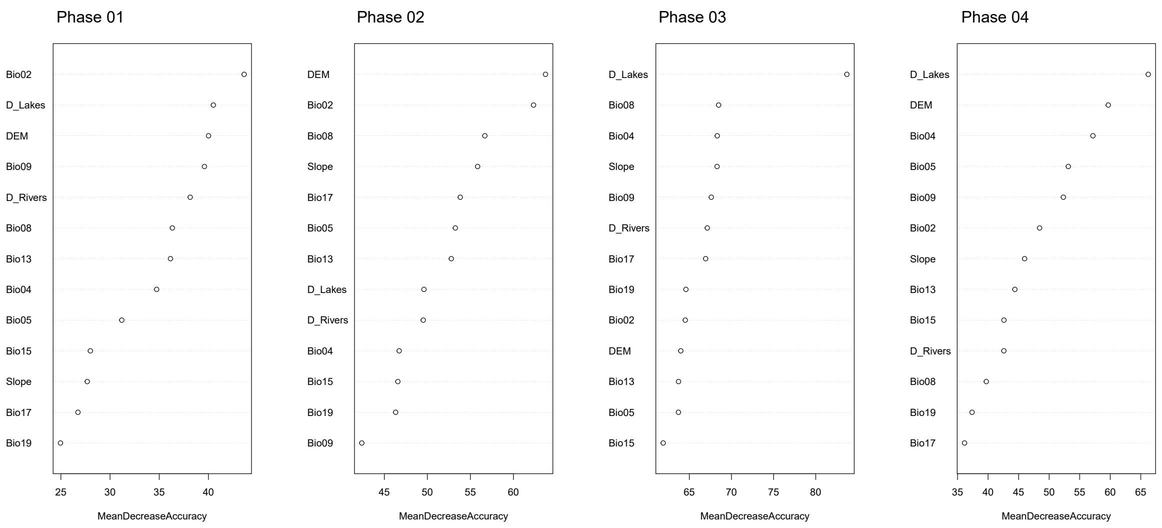

Supplementary Figure 4: Variable importance ranking for Random Forest models. Variable importance ranking as Mean Decrease Accuracy, computed by looking at how much the tree nodes, which use that variable, reduce the mean square errors estimated with the out-of-bag, across all the trees in the forest. From left to right: Phase 01, Phase 02, Phase 03 and Phase 04. Bio01: Annual Mean Temperature; Bio02: Mean Diurnal Range; Bio04: Temperature Seasonality; Bio05: Maximum Temperature of Warmest Month; Bio06: Minimum Temperature of Coldest Month; Bio07: Temperature Annual Range; Bio08: Mean Temperature of Wettest Quarter; Bio09: Mean Temperature of Driest Quarter; Bio10: Mean Temperature of Warmest Quarter; Bio11: Mean Temperature of Coldest Quarter; Bio12: Annual Precipitation; Bio13: Precipitation of Wettest Month; Bio14: Precipitation of Driest Month; Bio15: Precipitation Seasonality; Bio16: Precipitation of Wettest Quarter; Bio17: Precipitation of Driest Quarter; Bio18: Precipitation of Warmest Quarter; Bio19: Precipitation of Coldest Quarter; DEM: Digital Elevation Model; Slope: Slope; TRI: Terrain Ruggedness Index; D_Lakes: Distance from main lakes; D_Rivers: Distance from main rivers. Source data can be found on https://doi.org/10.5281/zenodo.14253277.

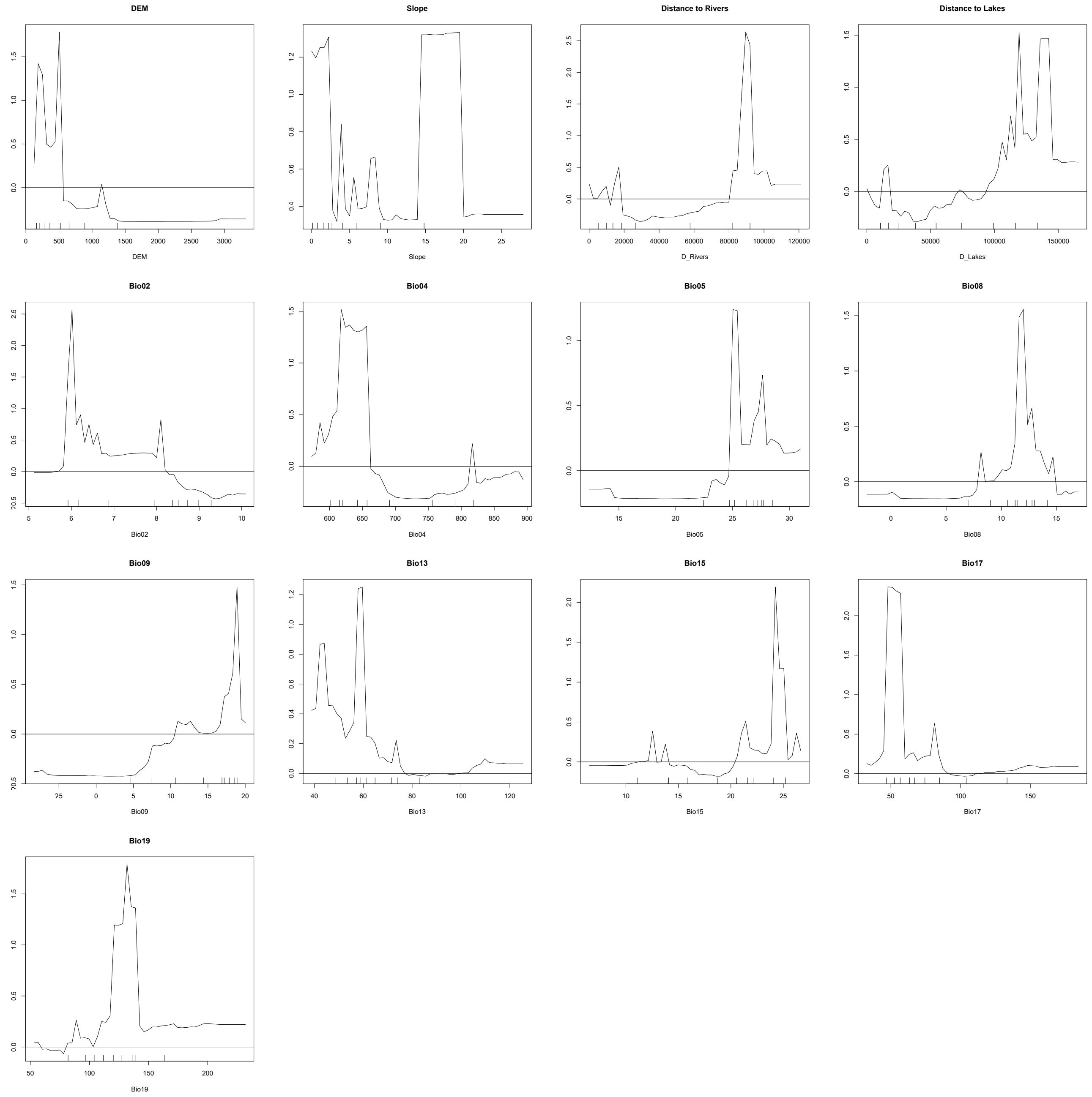

Supplementary Figure 5a: Partial Dependence Plots for Random Forest (RF) models Phase 01. The graphics indicate the influence (or marginal effect) of the specific class/range of values on the computed probability of site location. The Y axis of each plot indicates the 'Partial Dependence', thus positive values (above 0.0) on this axis show that sites are likely to be found for that value of the independent variable (x-axis), while negative values (below 0.0) indicate that sites are less likely to be found. Zero implies no average impact on site prediction according to the model. The hash marks at the bottom of each plot indicate the deciles of the distribution on the x axis (variable values). DEM: Digital Elevation Model; Slope: Slope; Bio02: Mean Diurnal Range; Bio04: Temperature Seasonality; Bio05: Maximum Temperature of Warmest Month; Bio08: Mean Temperature of Wettest Quarter; Bio09: Mean Temperature of Driest Quarter; Bio13: Precipitation of Wettest Month; Bio15: Precipitation Seasonality; Bio17: Precipitation of Driest Quarter; Bio19: Precipitation of Coldest Quarter. Source data can be found on https://doi.org/10.5281/zenodo.14253277.

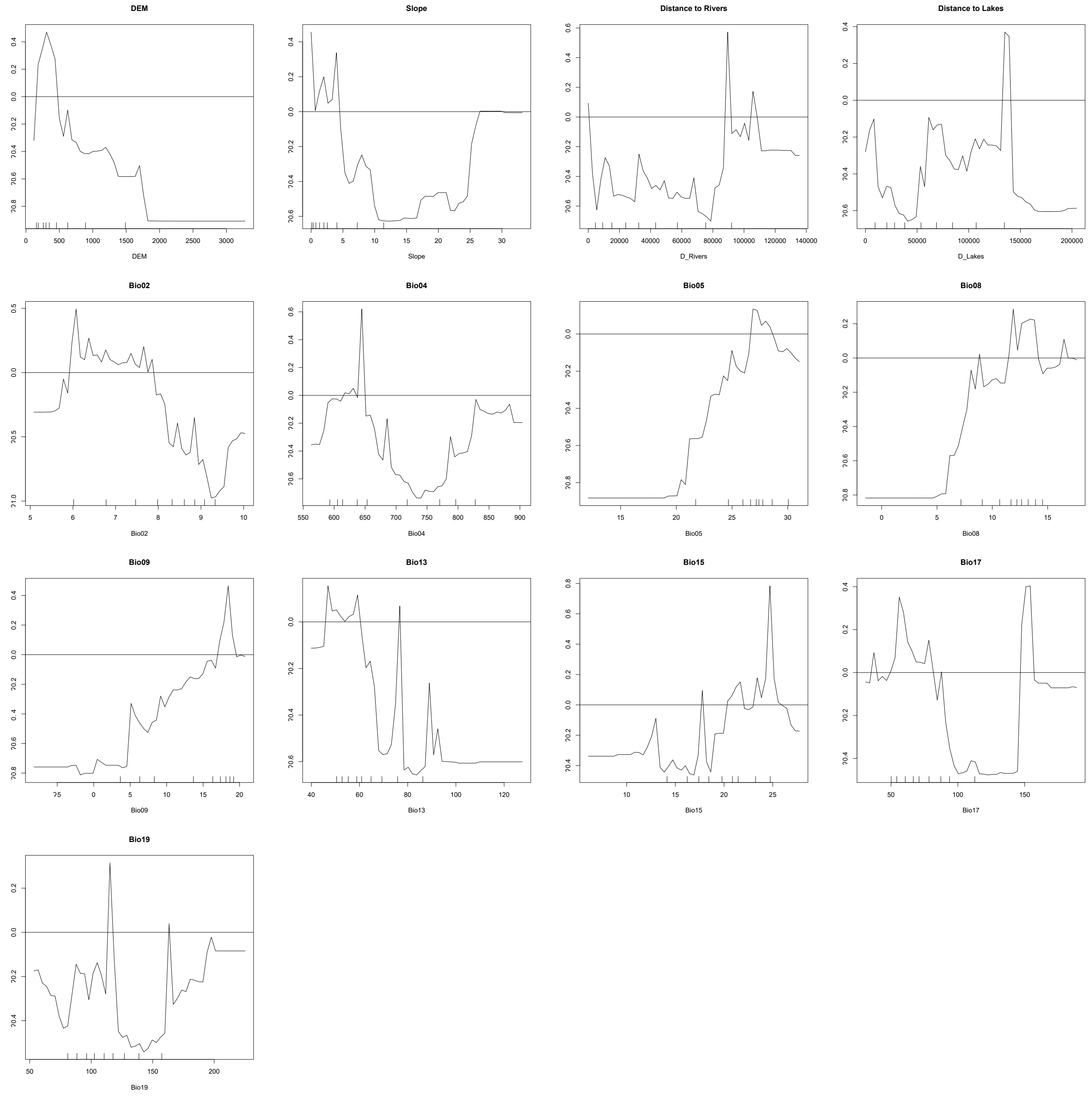

Supplementary Figure 5b: Partial Dependence Plots for Random Forest (RF) models Phase 02. The graphics indicate the influence (or marginal effect) of the specific class/range of values on the computed probability of site location. The Y axis of each plot indicates the 'Partial Dependence', thus positive values (above 0.0) on this axis show that sites are likely to be found for that value of the independent variable (x-axis), while negative values (below 0.0) indicate that sites are less likely to be found. Zero implies no average impact on site prediction according to the model. The hash marks at the bottom of each plot indicate the deciles of the distribution on the x axis (variable values). DEM: Digital Elevation Model; Slope: Slope; Bio02: Mean Diurnal Range; Bio04: Temperature Seasonality; Bio05: Maximum Temperature of Warmest Month; Bio08: Mean Temperature of Wettest Quarter; Bio09: Mean Temperature of Driest Quarter; Bio13: Precipitation of Wettest Month; Bio15: Precipitation Seasonality; Bio17: Precipitation of Driest Quarter; Bio19: Precipitation of Coldest Quarter. Source data can be found on https://doi.org/10.5281/zenodo.14253277.

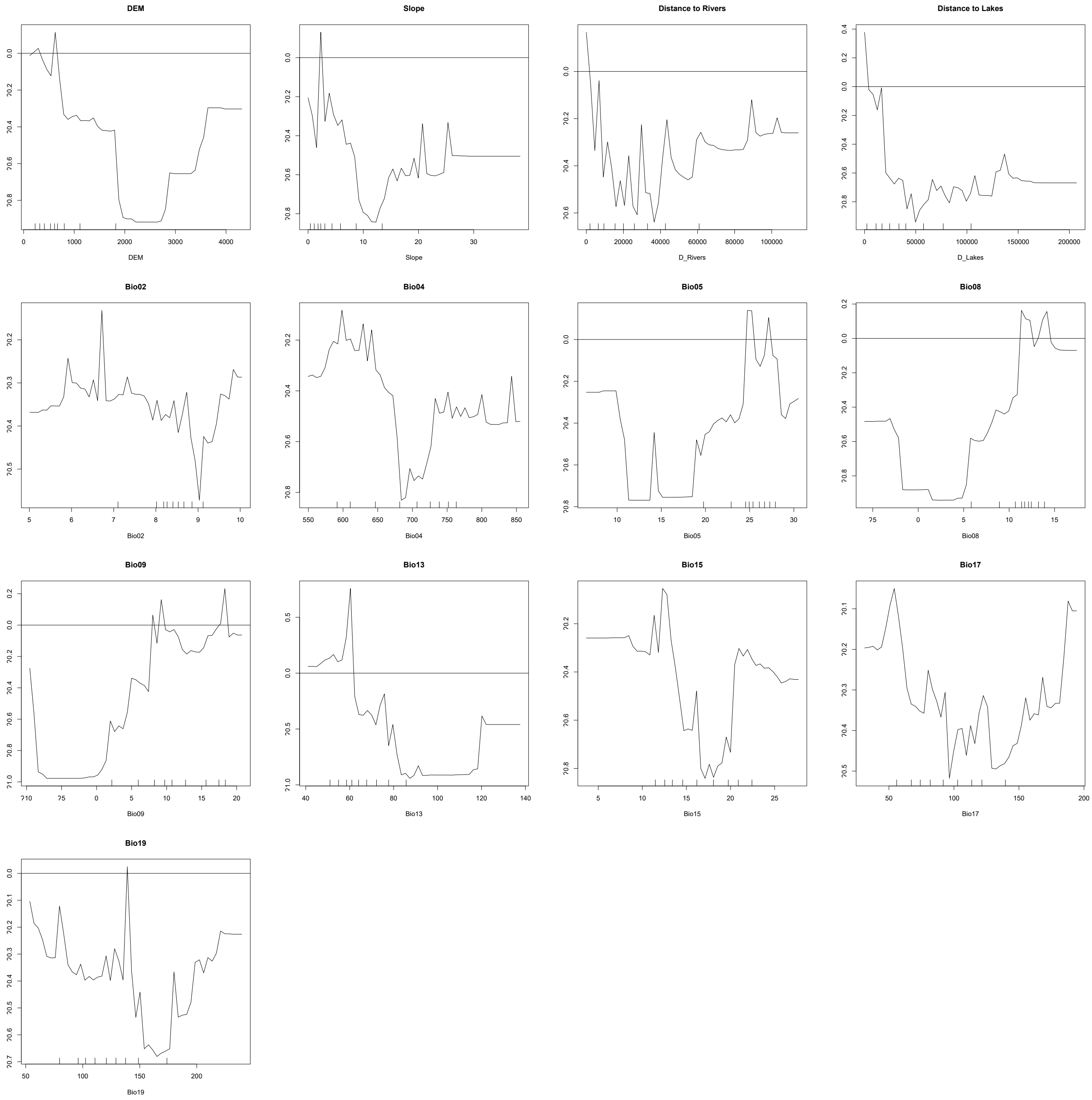

Supplementary Figure 5c: Partial Dependence Plots for Random Forest (RF) models Phase 03. The graphics indicate the influence (or marginal effect) of the specific class/range of values on the computed probability of site location. The Y axis of each plot indicates the 'Partial Dependence', thus positive values (above 0.0) on this axis show that sites are likely to be found for that value of the independent variable (x-axis), while negative values (below 0.0) indicate that sites are less likely to be found. Zero implies no average impact on site prediction according to the model. The hash marks at the bottom of each plot indicate the deciles of the distribution on the x axis (variable values). DEM: Digital Elevation Model; Slope: Slope; Bio02: Mean Diurnal Range; Bio04: Temperature Seasonality; Bio05: Maximum Temperature of Warmest Month; Bio08: Mean Temperature of Wettest Quarter; Bio09: Mean Temperature of Driest Quarter; Bio13: Precipitation of Wettest Month; Bio15: Precipitation Seasonality; Bio17: Precipitation of Driest Quarter; Bio19: Precipitation of Coldest Quarter. Source data can be found on https://doi.org/10.5281/zenodo.14253277.

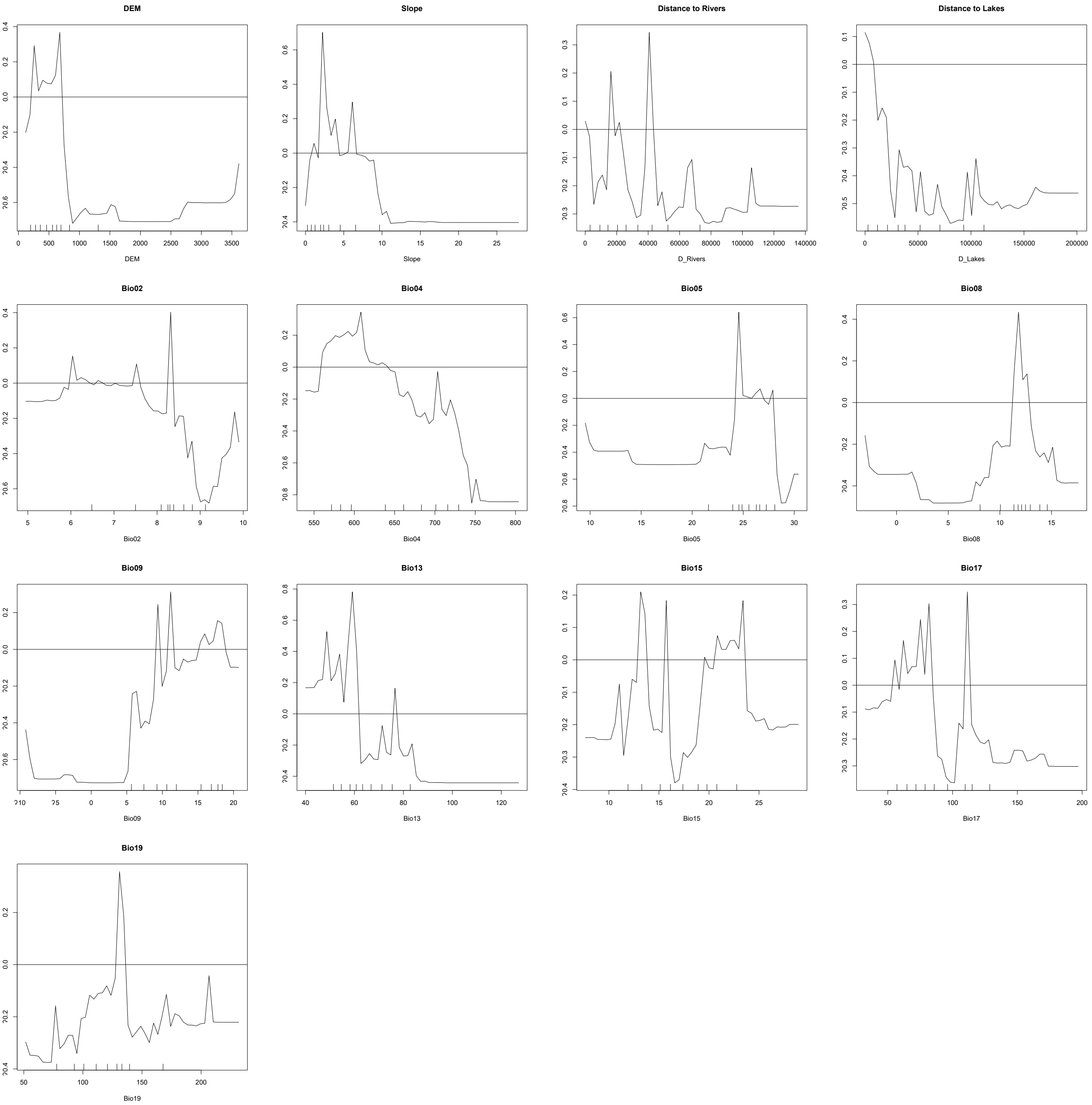

Supplementary Figure 5d: Partial Dependence Plots for Random Forest (RF) models Phase 04. The graphics indicate the influence (or marginal effect) of the specific class/range of values on the computed probability of site location. The Y axis of each plot indicates the 'Partial Dependence', thus positive values (above 0.0) on this axis show that sites are likely to be found for that value of the independent variable (x-axis), while negative values (below 0.0) indicate that sites are less likely to be found. Zero implies no average impact on site prediction according to the model. The hash marks at the bottom of each plot indicate the deciles of the distribution on the x axis (variable values). DEM: Digital Elevation Model; Slope: Slope; Bio02: Mean Diurnal Range; Bio04: Temperature Seasonality; Bio05: Maximum Temperature of Warmest Month; Bio08: Mean Temperature of Wettest Quarter; Bio09: Mean Temperature of Driest Quarter; Bio13: Precipitation of Wettest Month; Bio15: Precipitation Seasonality; Bio17: Precipitation of Driest Quarter; Bio19: Precipitation of Coldest Quarter. Source data can be found on https://doi.org/10.5281/zenodo.14253277.

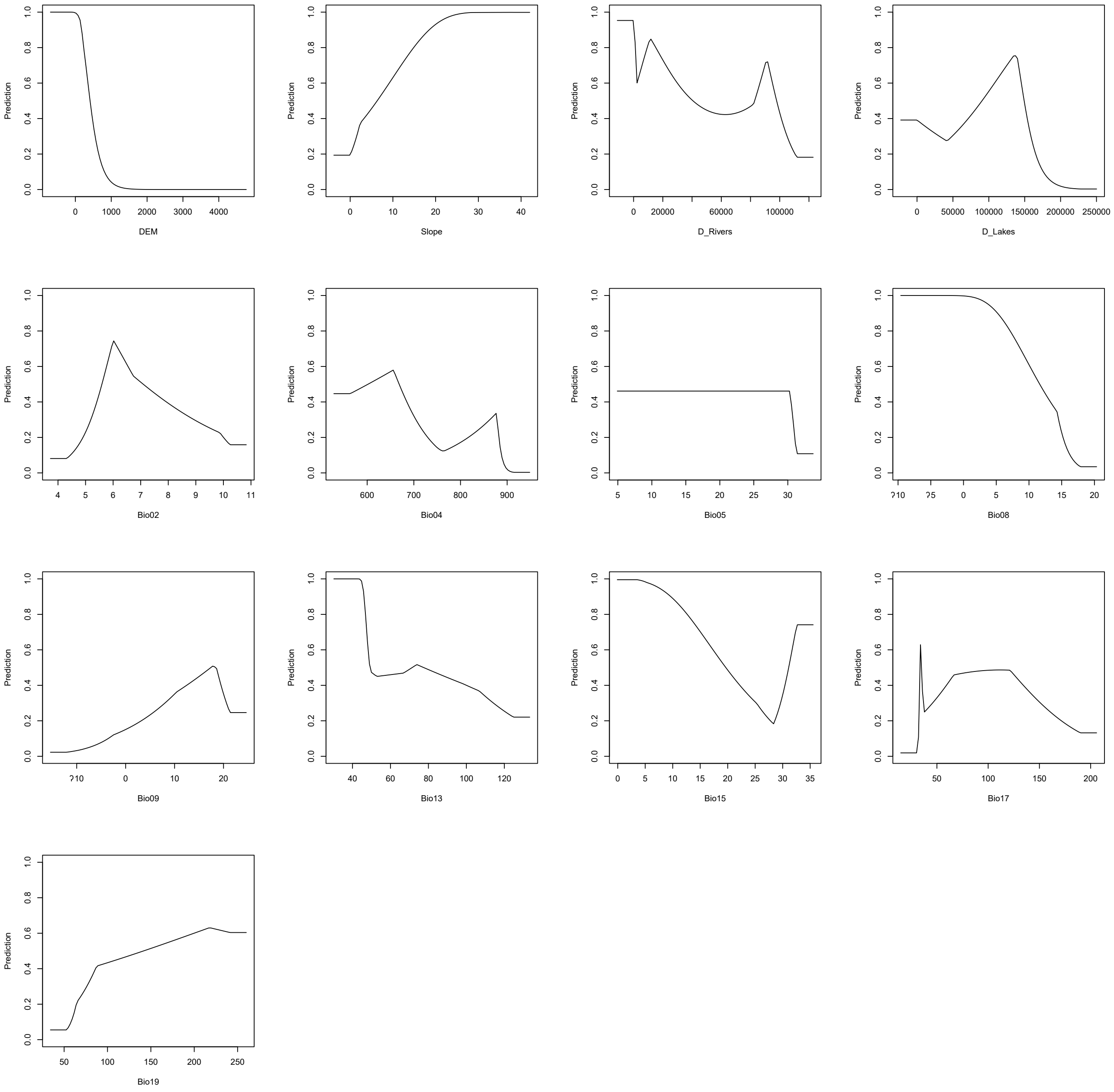

Supplementary Figure 6a: Response Plots for Maximum Entropy (MaxEnt) models Phase 01. Each response plot indicates the role of a particular variable depicted as a response curve showing the predicted relative occurrence rate (ROR; y axis) against the values of that predictor (variable; x axis). DEM: Digital Elevation Model; Slope: Slope; D_Lakes: Distance from main lakes; D_Rivers: Distance from main rivers; Bio02: Mean Diurnal Range; Bio04: Temperature Seasonality; Bio05: Maximum Temperature of Warmest Month; Bio08: Mean Temperature of Wettest Quarter; Bio09: Mean Temperature of Driest Quarter; Bio13: Precipitation of Wettest Month; Bio15: Precipitation Seasonality; Bio17: Precipitation of Driest Quarter; Bio19: Precipitation of Coldest Quarter. Source data can be found on https://doi.org/10.5281/zenodo.14253277.

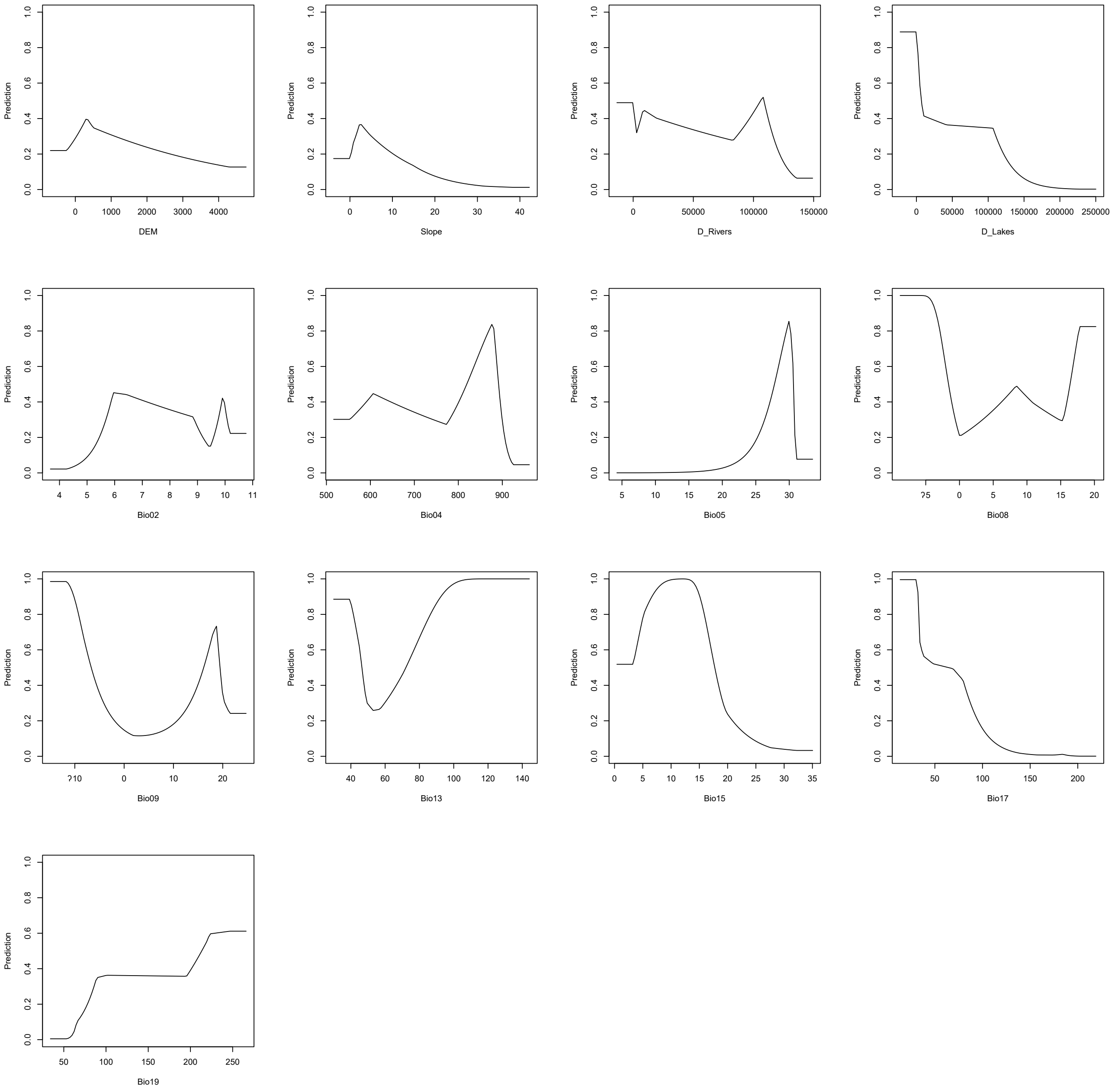

Supplementary Figure 6b: Response Plots for Maximum Entropy (MaxEnt) models Phase 02. Each response plot indicates the role of a particular variable depicted as a response curve showing the predicted relative occurrence rate (ROR; y axis) against the values of that predictor (variable; x axis). DEM: Digital Elevation Model; Slope: Slope; D_Lakes: Distance from main lakes; D_Rivers: Distance from main rivers; Bio02: Mean Diurnal Range; Bio04: Temperature Seasonality; Bio05: Maximum Temperature of Warmest Month; Bio08: Mean Temperature of Wettest Quarter; Bio09: Mean Temperature of Driest Quarter; Bio13: Precipitation of Wettest Month; Bio15: Precipitation Seasonality; Bio17: Precipitation of Driest Quarter; Bio19: Precipitation of Coldest Quarter. Source data can be found on https://doi.org/10.5281/zenodo.14253277.

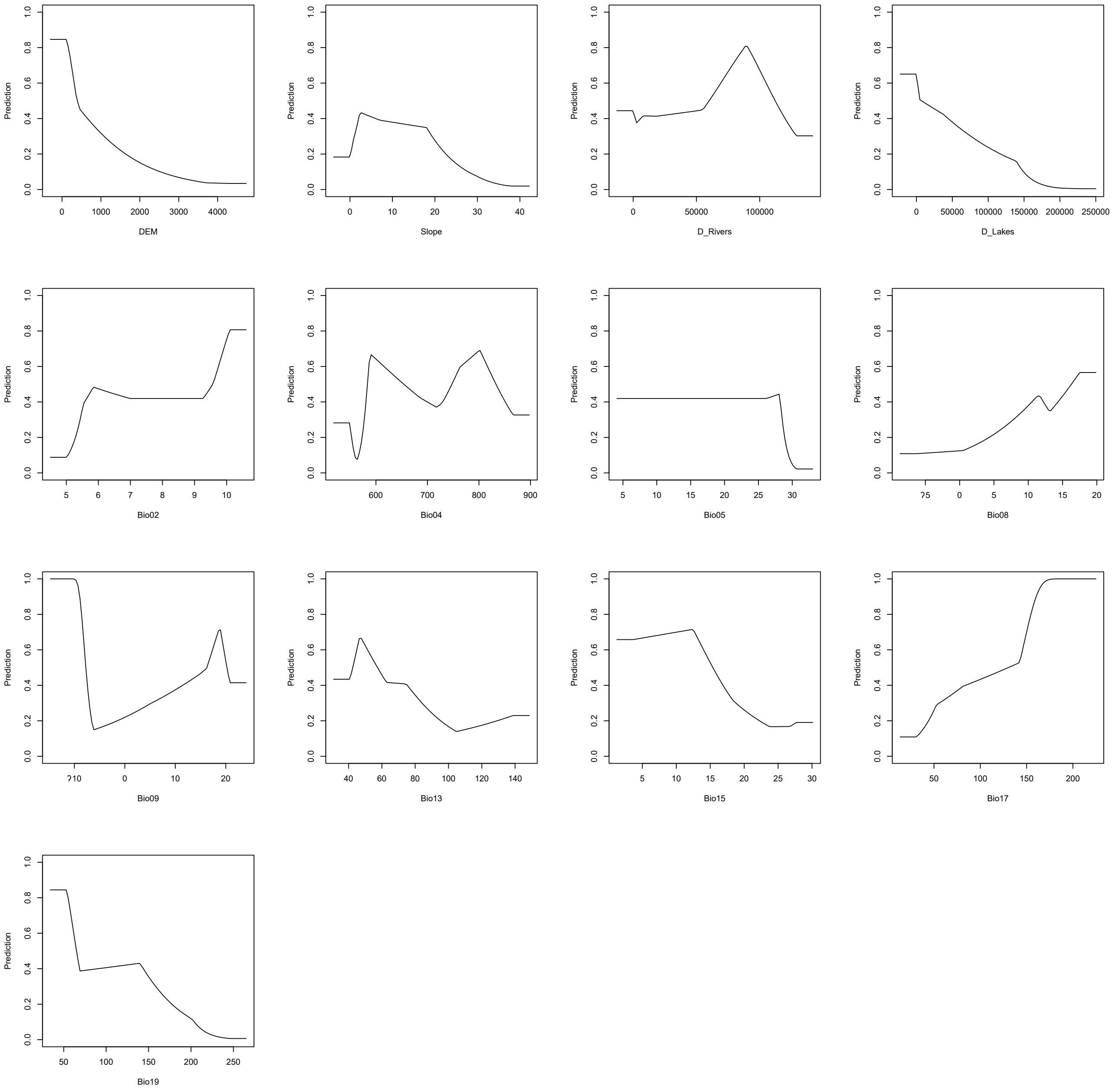

Supplementary Figure 6c: Response Plots for Maximum Entropy (MaxEnt) models Phase 03. Each response plot indicates the role of a particular variable depicted as a response curve showing the predicted relative occurrence rate (ROR; y axis) against the values of that predictor (variable; x axis). DEM: Digital Elevation Model; Slope: Slope; D_Lakes: Distance from main lakes; D_Rivers: Distance from main rivers; Bio02: Mean Diurnal Range; Bio04: Temperature Seasonality; Bio05: Maximum Temperature of Warmest Month; Bio08: Mean Temperature of Wettest Quarter; Bio09: Mean Temperature of Driest Quarter; Bio13: Precipitation of Wettest Month; Bio15: Precipitation Seasonality; Bio17: Precipitation of Driest Quarter; Bio19: Precipitation of Coldest Quarter. Source data can be found on https://doi.org/10.5281/zenodo.14253277.

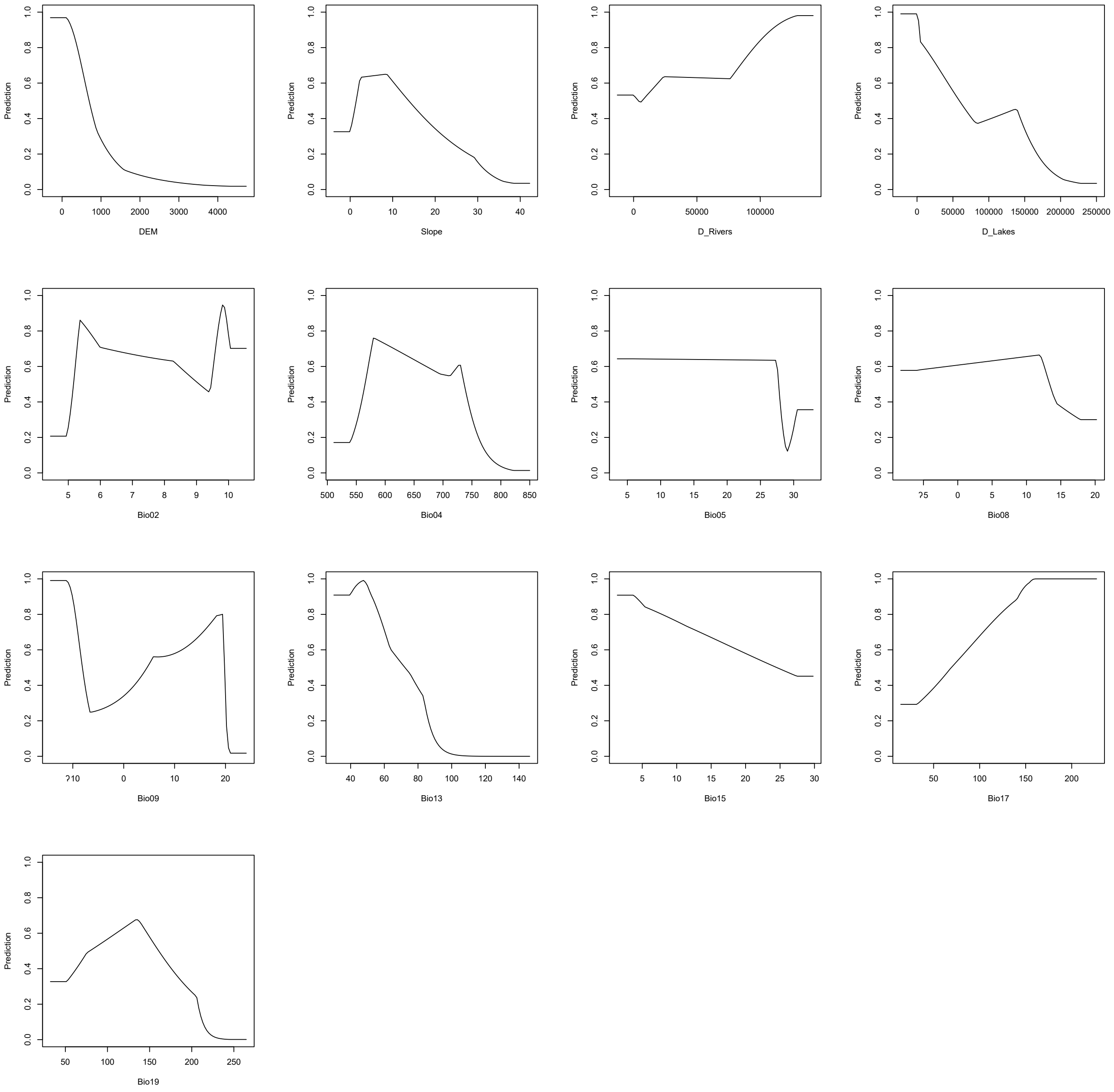

Supplementary Figure 6d: Response Plots for Maximum Entropy (MaxEnt) models Phase 04. Each response plot indicates the role of a particular variable depicted as a response curve showing the predicted relative occurrence rate (ROR; y axis) against the values of that predictor (variable; x axis). DEM: Digital Elevation Model; Slope: Slope; D_Lakes: Distance from main lakes; D_Rivers: Distance from main rivers; Bio02: Mean Diurnal Range; Bio04: Temperature Seasonality; Bio05: Maximum Temperature of Warmest Month; Bio08: Mean Temperature of Wettest Quarter; Bio09: Mean Temperature of Driest Quarter; Bio13: Precipitation of Wettest Month; Bio15: Precipitation Seasonality; Bio17: Precipitation of Driest Quarter; Bio19: Precipitation of Coldest Quarter. Source data can be found on https://doi.org/10.5281/zenodo.14253277.

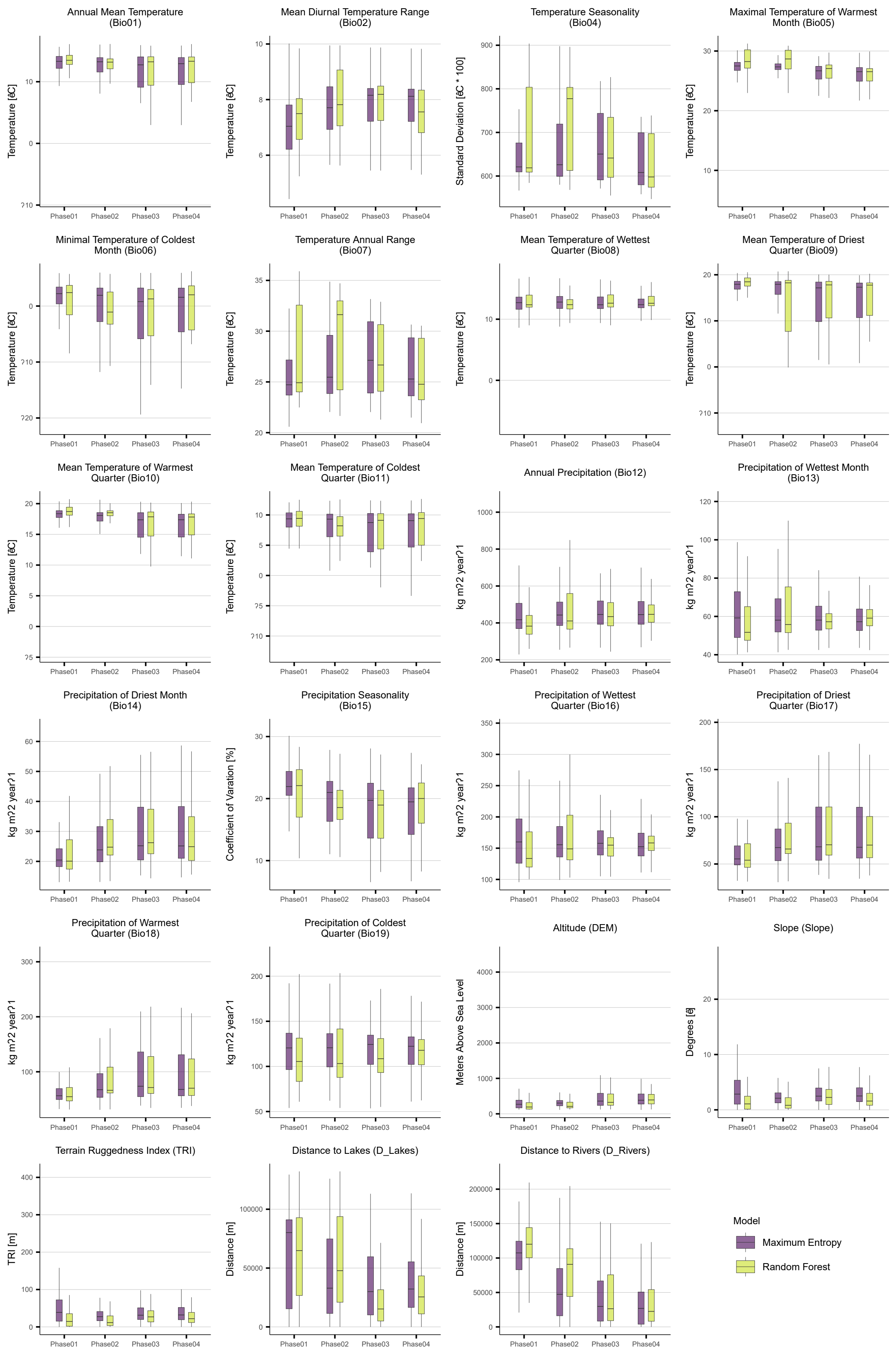

Supplementary Figure 7: Distribution of predicted high suitability areas (≥ 0.75) modeled using Maximum Entropy and Random Forest over paleoclimate and environmental variables per Phase. Boxplots in each panel show medians, first and third quartiles (hinges), minimum and maximum values no further than 1.5*IQR from the hinge where IQR is the inter- quartile range (whiskers). Phase 01: RF: n=14270, MaxEnt: n=9493; Phase 02: RF: n=6770, MaxEnt: n=19506; Phase 03: RF: n=3274, MaxEnt: n=20761; Phase 04: RF: n=12090, MaxEnt: n=20708. Source data can be found on https://doi.org/10.5281/zenodo.14253277.

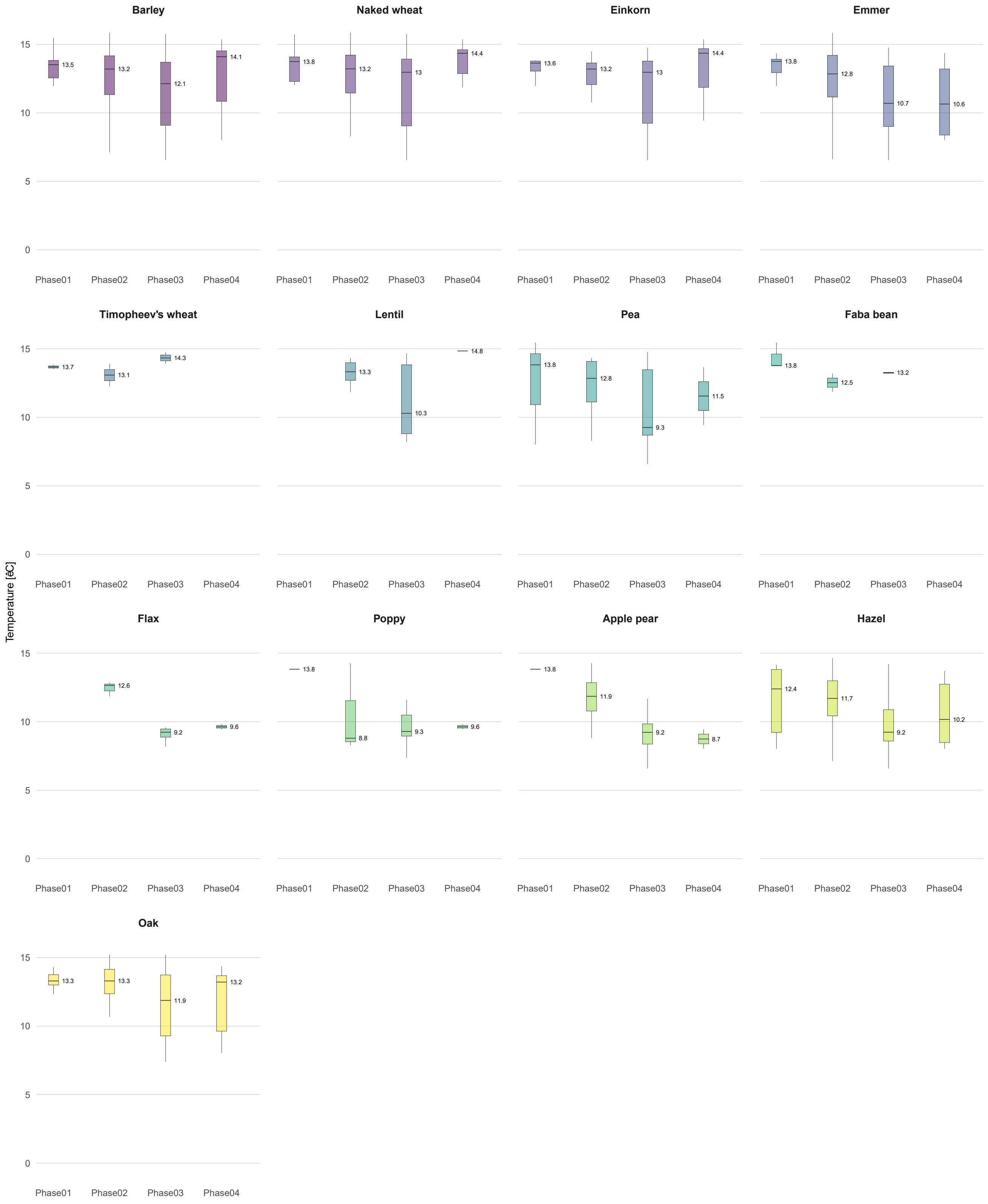

Supplementary Figure 8a: Distribution of crop occurrences over Annual Mean Temperature (Bio01) per Phase. The boxplots show medians, first and third quartiles (hinges), minimum and maximum values no further than 1.5*IQR from the hinge where IQR is the inter-quartile range (whiskers). Details about sample numbers are provided in Supplementary Table 3. Source data can be found on https://doi.org/10.5281/zenodo.14253277.

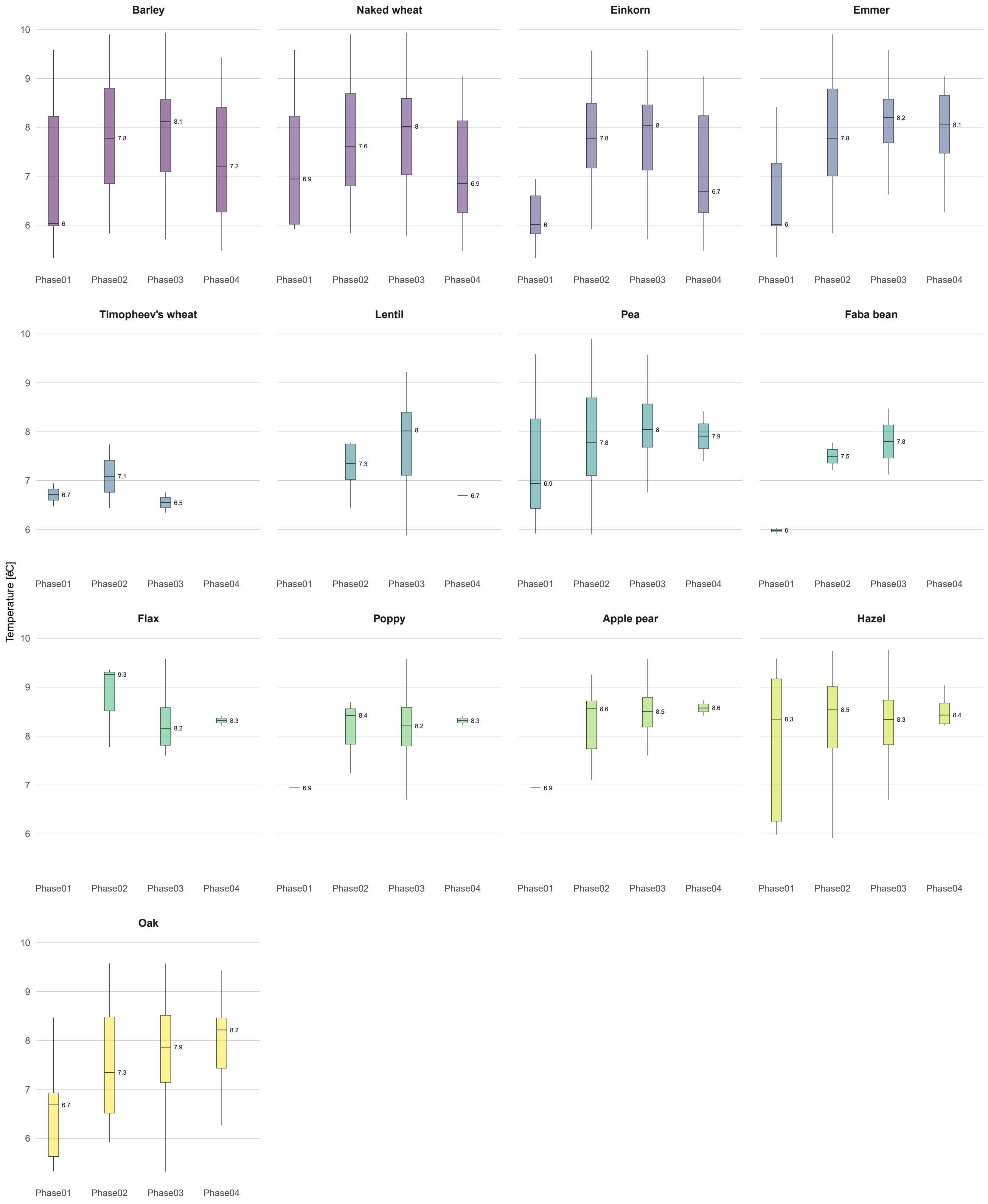

Supplementary Figure 8b: Distribution of crop occurrences over Mean Diurnal Temperature Range (Bio02) per Phase. The boxplots show medians, first and third quartiles (hinges), minimum and maximum values no further than 1.5*IQR from the hinge where IQR is the inter- quartile range (whiskers). Details about sample numbers are provided in Supplementary Table 3. Source data can be found on https://doi.org/10.5281/zenodo.14253277.

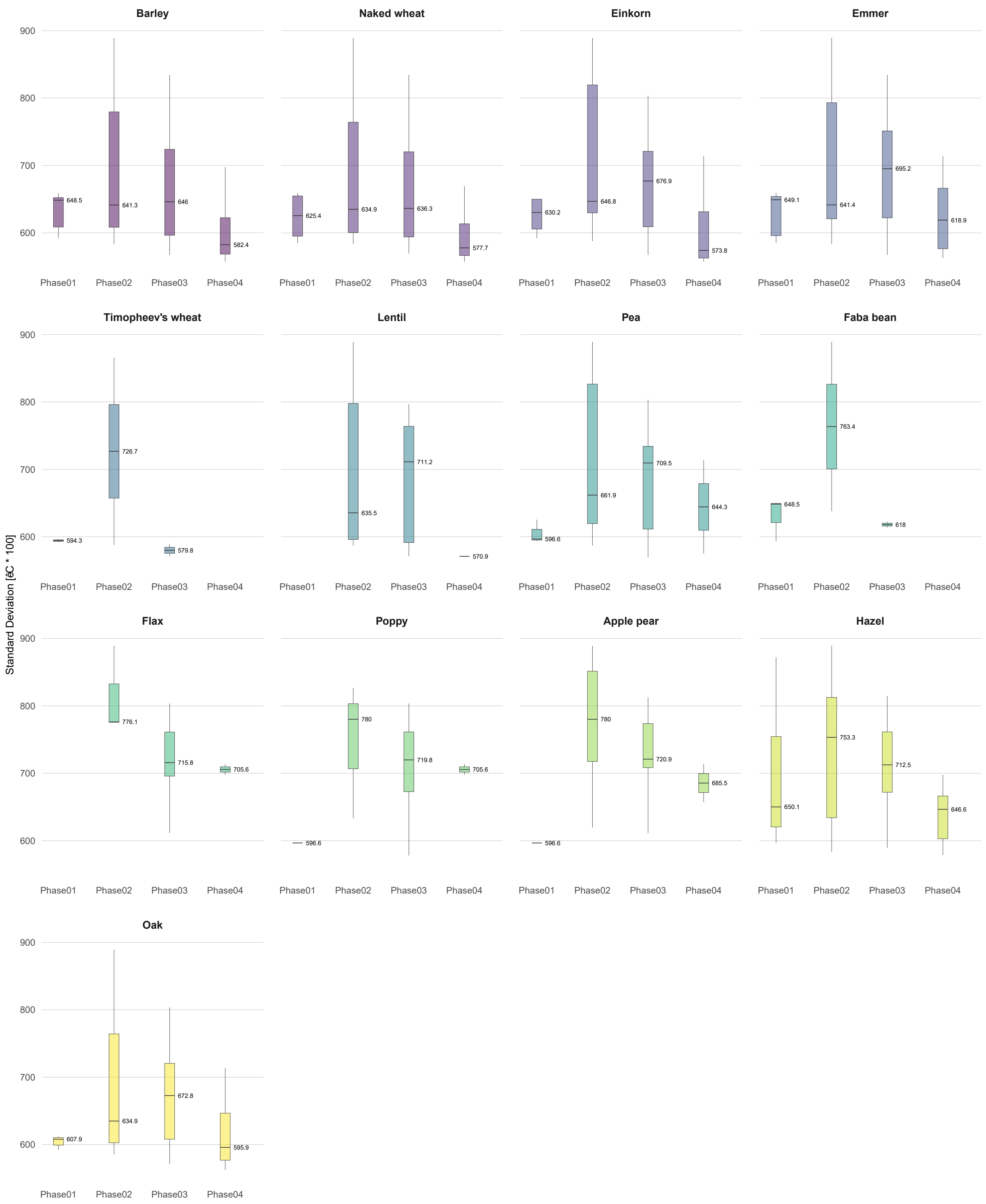

Supplementary Figure 8c: Distribution of crop occurrences over Temperature Seasonality (Bio04) per Phase. The boxplots show medians, first and third quartiles (hinges), minimum and maximum values no further than 1.5*IQR from the hinge where IQR is the inter-quartile range (whiskers). Details about sample numbers are provided in Supplementary Table 3. Source data can be found on https://doi.org/10.5281/zenodo.14253277.

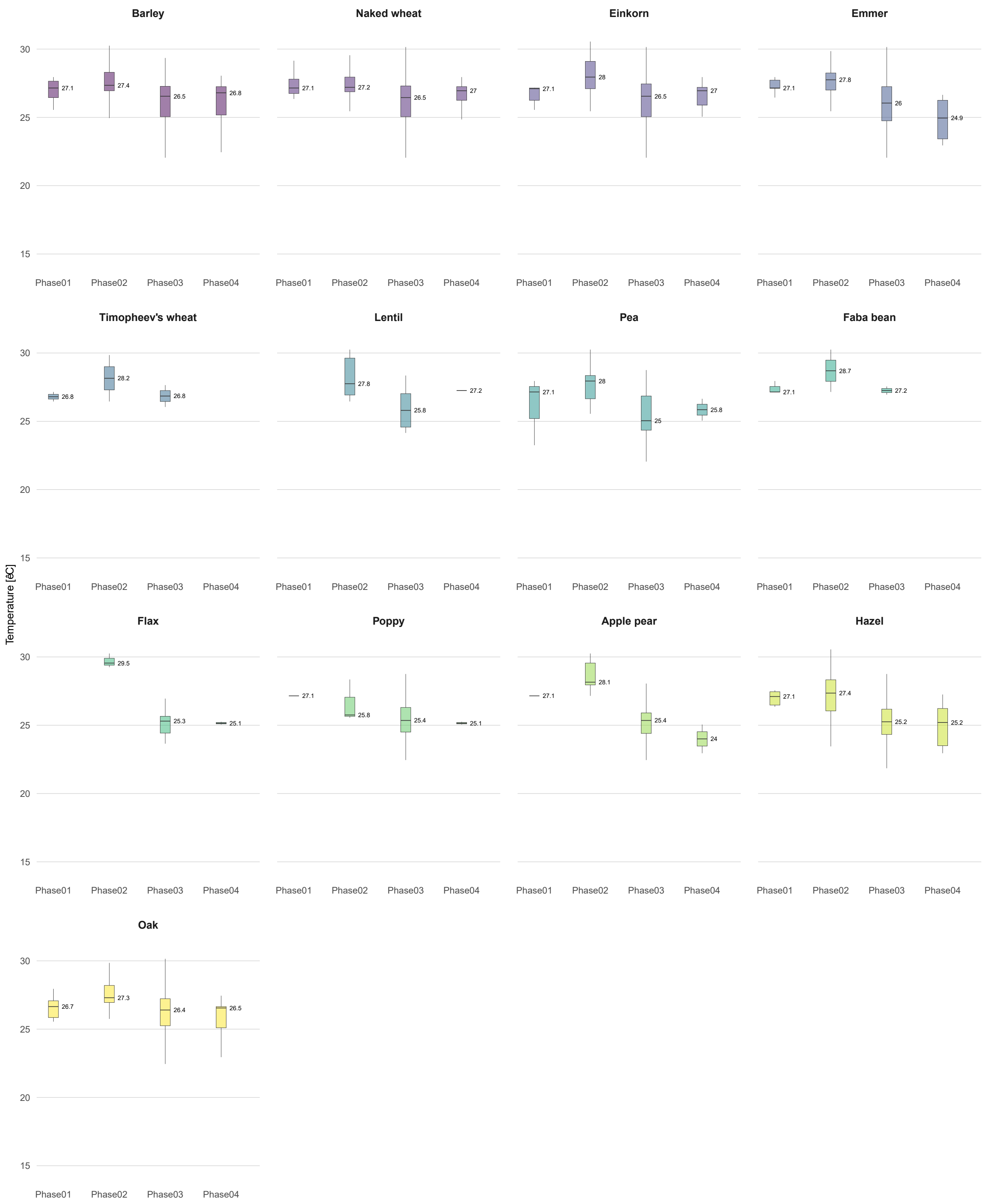

Supplementary Figure 8d: Distribution of crop occurrences over Max. Temperature of Warmest Month (Bio05) per Phase. The boxplots show medians, first and third quartiles (hinges), minimum and maximum values no further than 1.5*IQR from the hinge where IQR is the inter- quartile range (whiskers). Details about sample numbers are provided in Supplementary Table 3. Source data can be found on https://doi.org/10.5281/zenodo.14253277.

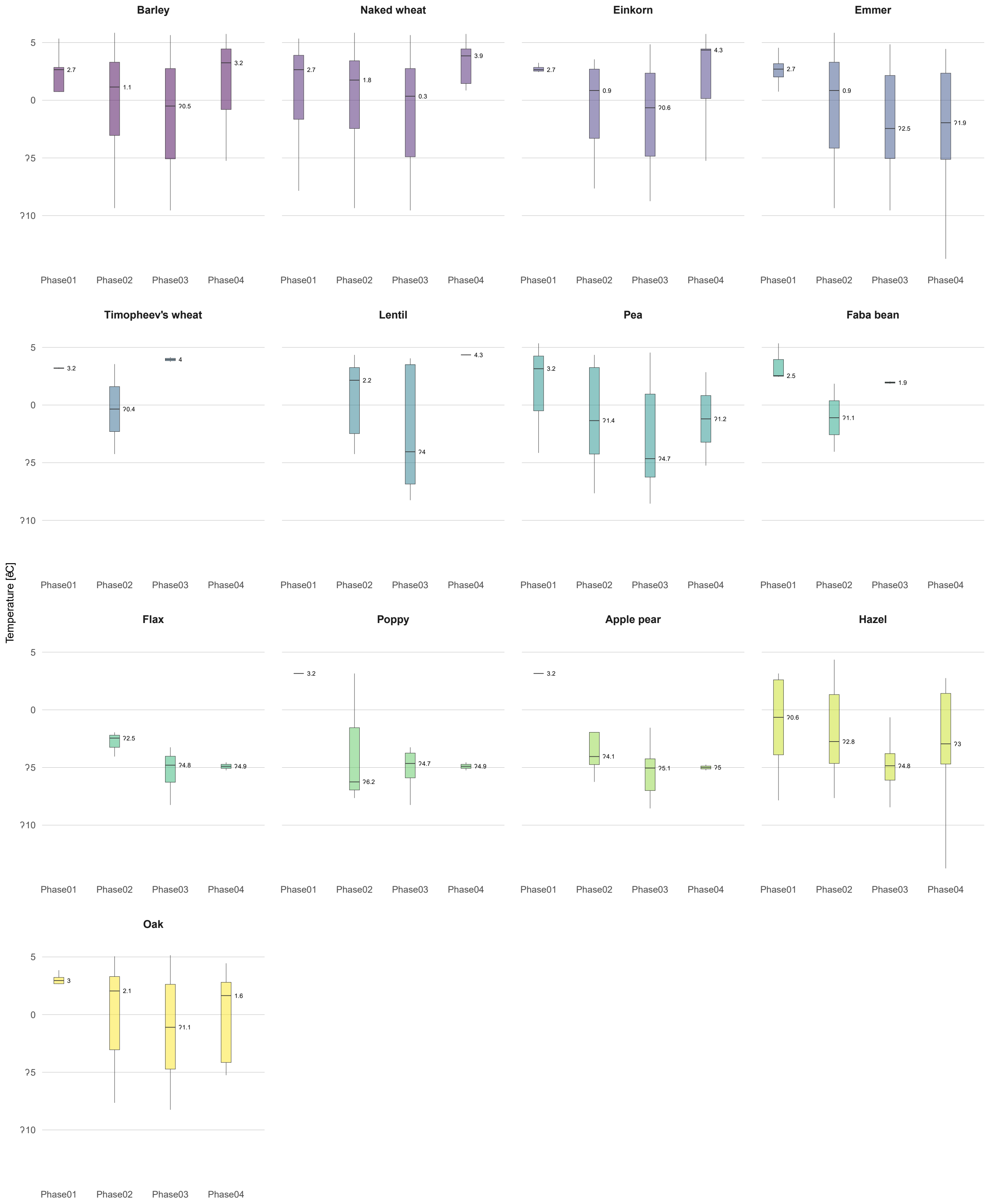

Supplementary Figure 8e: Distribution of crop occurrences over Min. Temperature of Coldest Month (Bio06) per Phase. The boxplots show medians, first and third quartiles (hinges), minimum and maximum values no further than 1.5*IQR from the hinge where IQR is the inter- quartile range (whiskers). Details about sample numbers are provided in Supplementary Table 3. Source data can be found on https://doi.org/10.5281/zenodo.14253277.

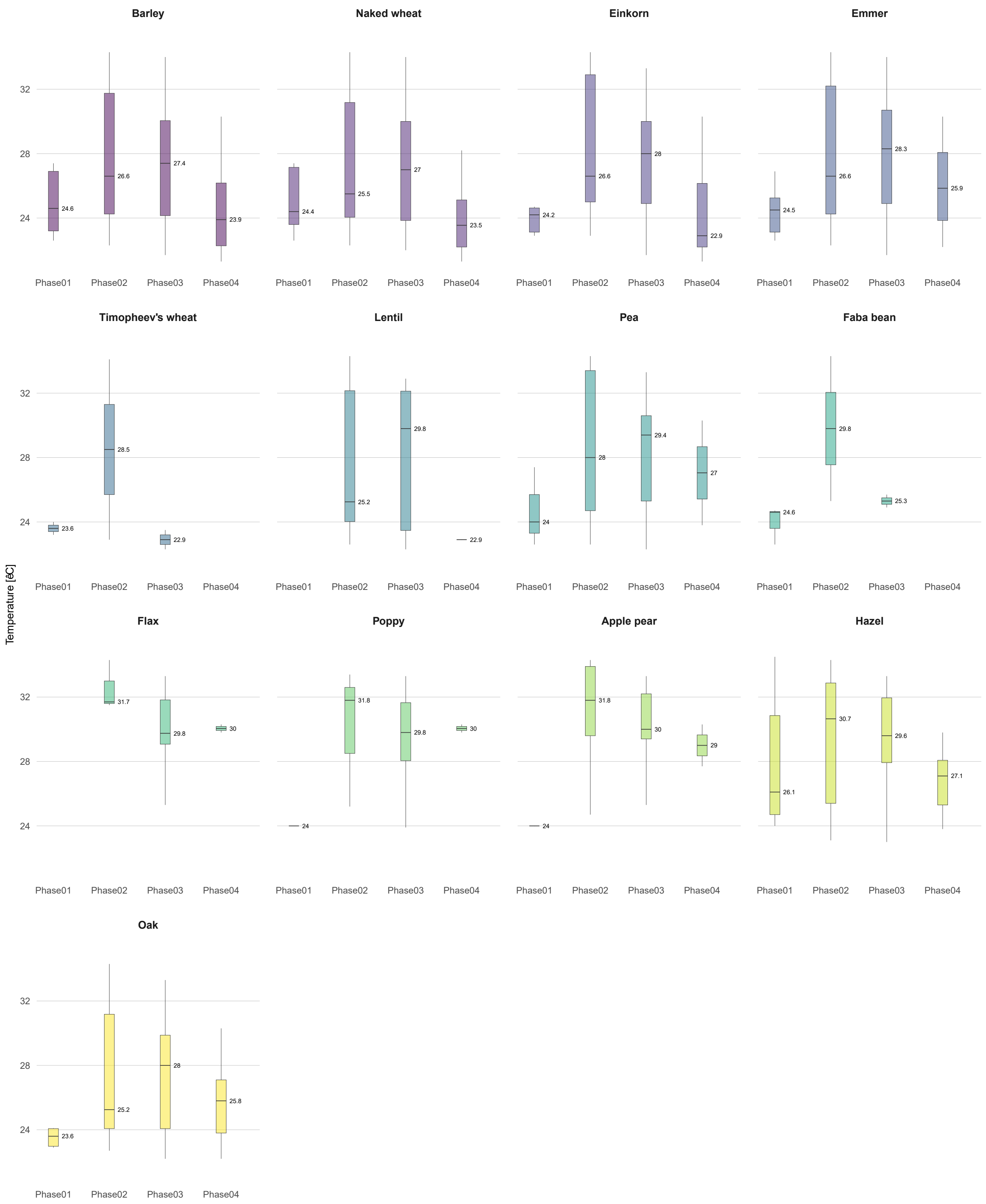

Supplementary Figure 8f: Distribution of crop occurrences over Temperature Annual Range (Bio07) per Phase. The boxplots show medians, first and third quartiles (hinges), minimum and maximum values no further than 1.5*IQR from the hinge where IQR is the inter-quartile range (whiskers). Details about sample numbers are provided in Supplementary Table 3. Source data can be found on https://doi.org/10.5281/zenodo.14253277.

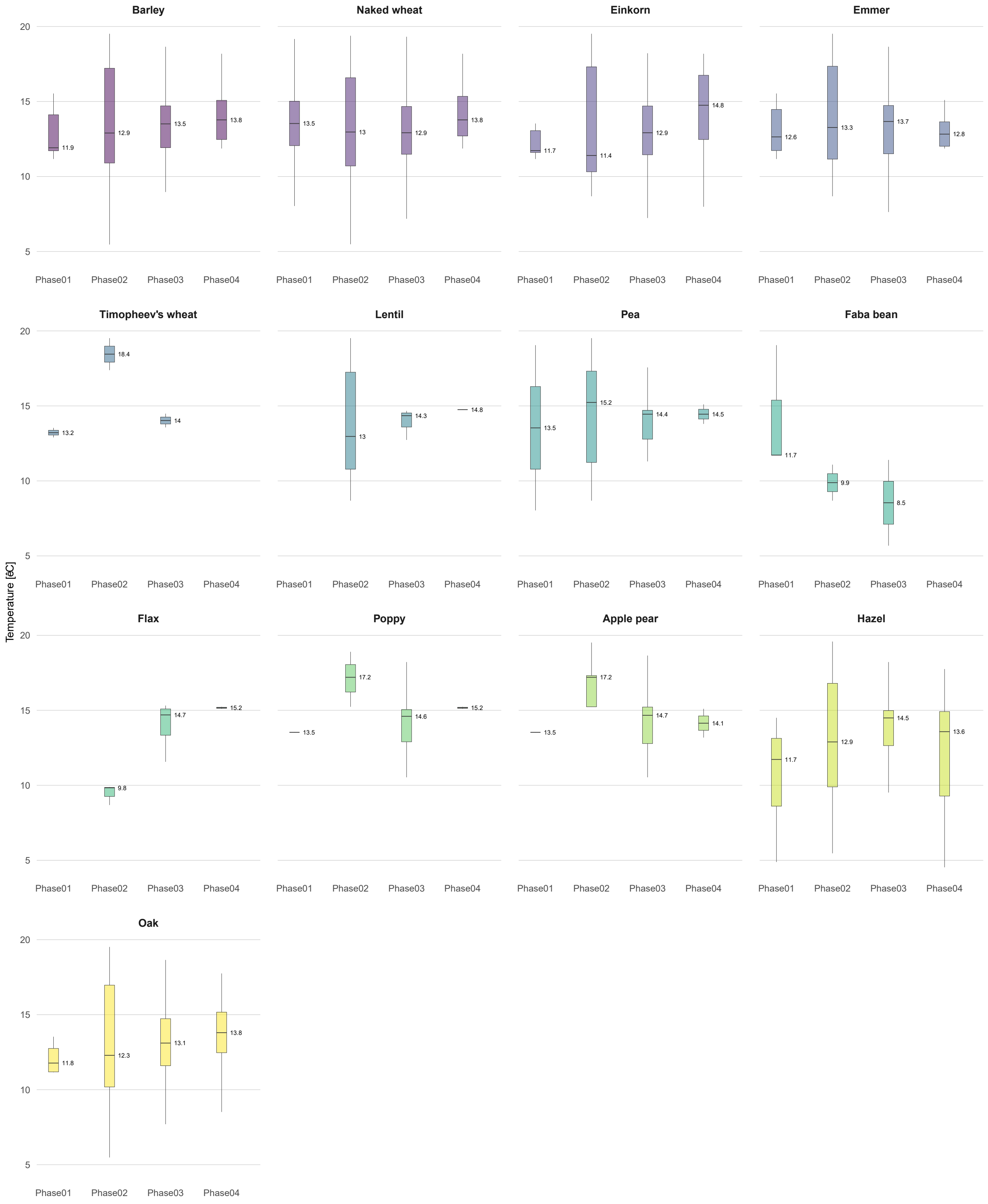

Supplementary Figure 8g: Distribution of crop occurrences over Mean Temperature of Wettest Quarter (Bio08) per Phase. The boxplots show medians, first and third quartiles (hinges), minimum and maximum values no further than 1.5*IQR from the hinge where IQR is the inter- quartile range (whiskers). Details about sample numbers are provided in Supplementary Table 3. Source data can be found on https://doi.org/10.5281/zenodo.14253277.

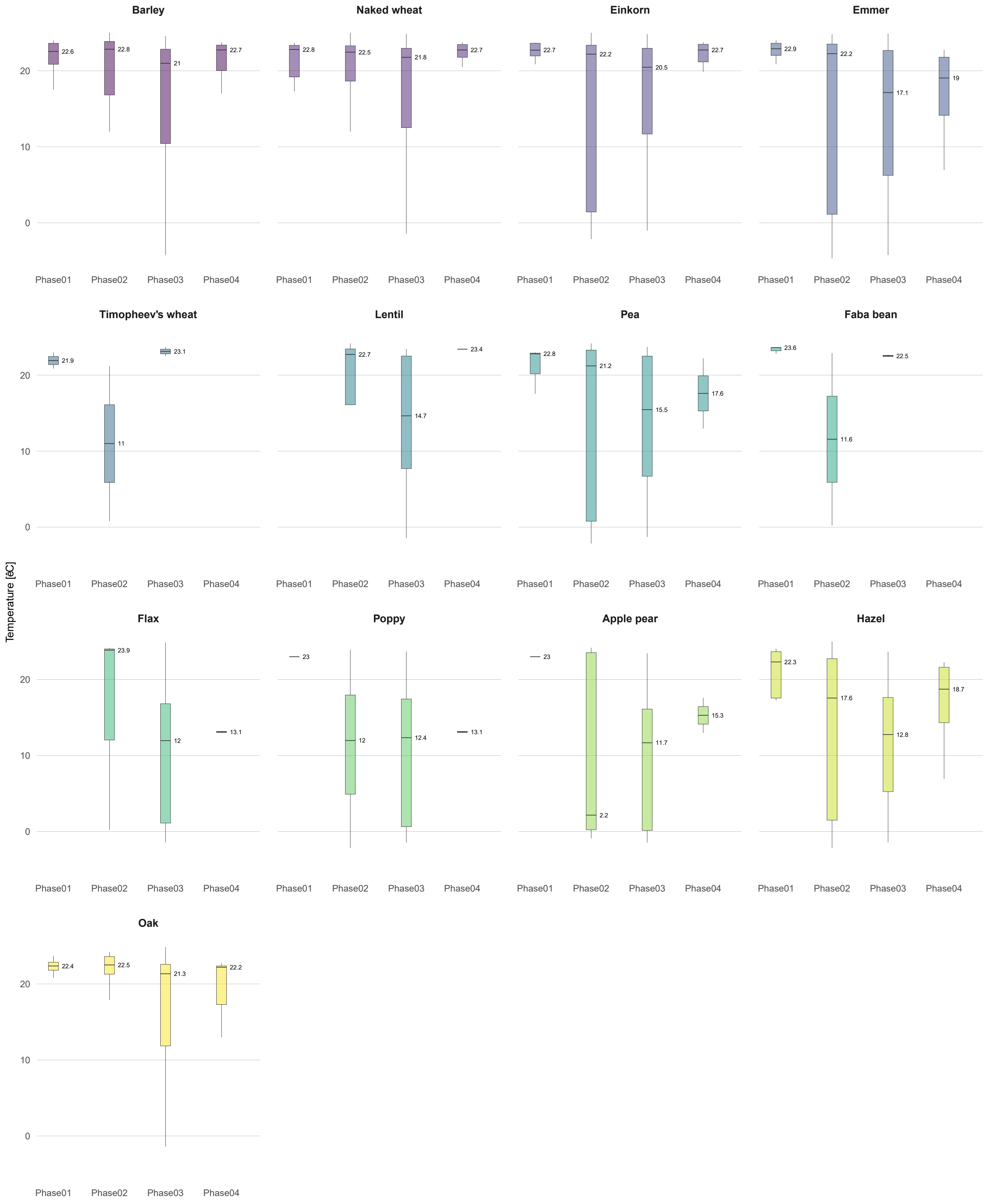

Supplementary Figure 8h: Distribution of crop occurrences over Mean Temperature of Driest Quarter (Bio09) per Phase. The boxplots show medians, first and third quartiles (hinges), minimum and maximum values no further than 1.5*IQR from the hinge where IQR is the inter- quartile range (whiskers). Details about sample numbers are provided in Supplementary Table 3. Source data can be found on https://doi.org/10.5281/zenodo.14253277.

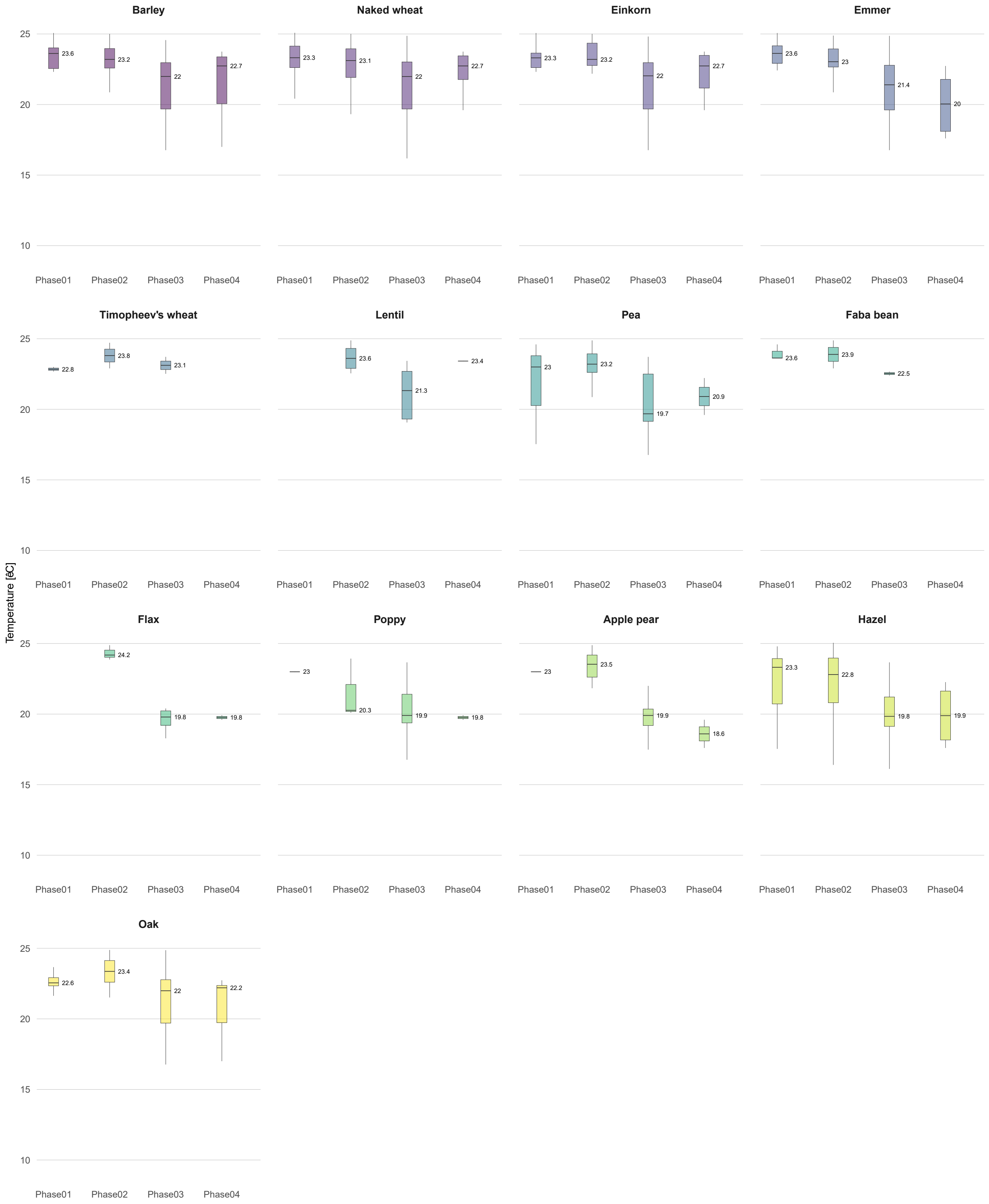

Supplementary Figure 8i: Distribution of crop occurrences over Mean Temperature of Warmest Quarter (Bio10) per Phase. The boxplots show medians, first and third quartiles (hinges), minimum and maximum values no further than 1.5*IQR from the hinge where IQR is the inter- quartile range (whiskers). Details about sample numbers are provided in Supplementary Table 3. Source data can be found on https://doi.org/10.5281/zenodo.14253277.

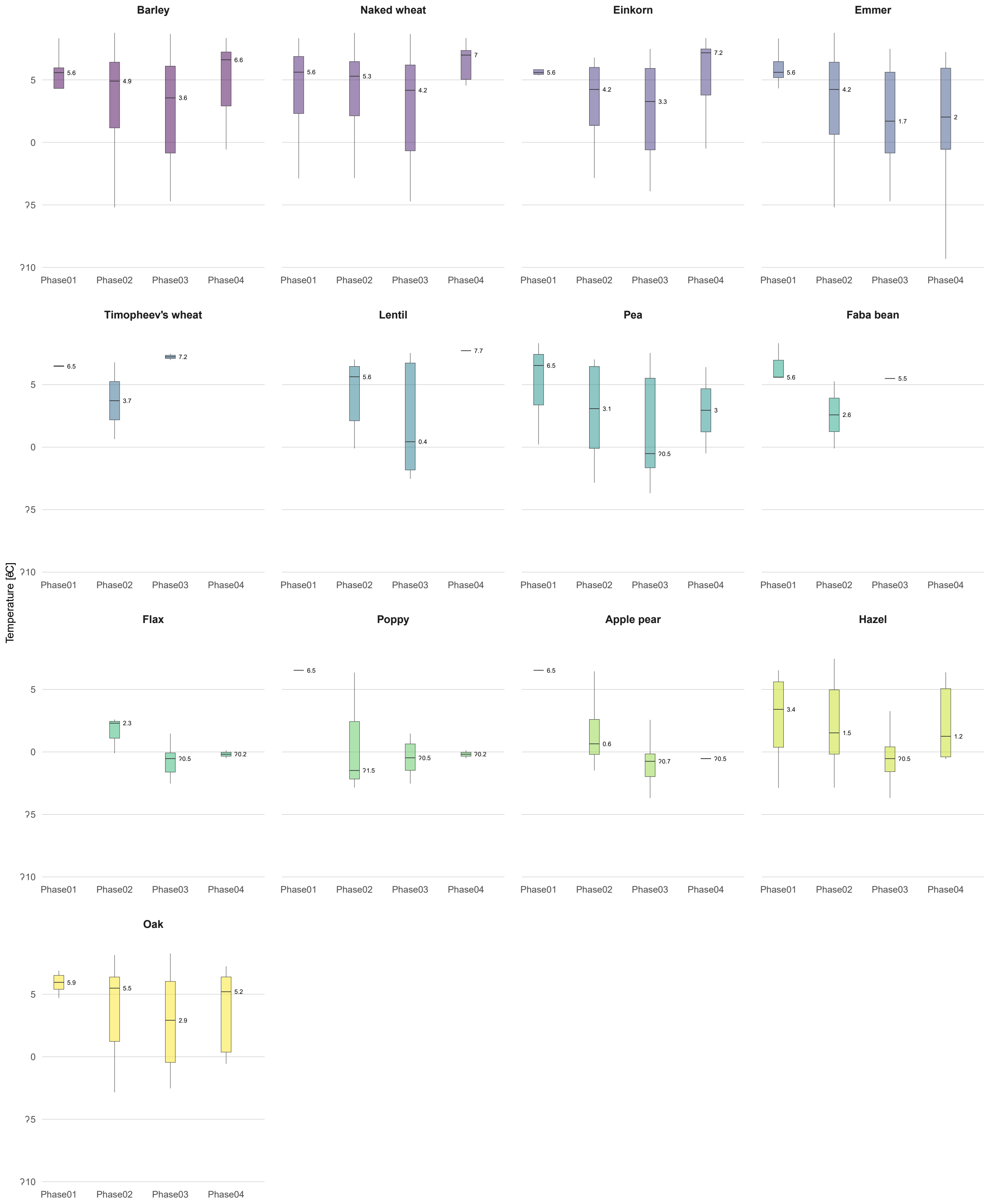

Supplementary Figure 8j: Distribution of crop occurrences over Mean Temperature of Coldest Quarter (Bio11) per Phase. The boxplots show medians, first and third quartiles (hinges), minimum and maximum values no further than 1.5*IQR from the hinge where IQR is the inter- quartile range (whiskers). Details about sample numbers are provided in Supplementary Table 3. Source data can be found on https://doi.org/10.5281/zenodo.14253277.

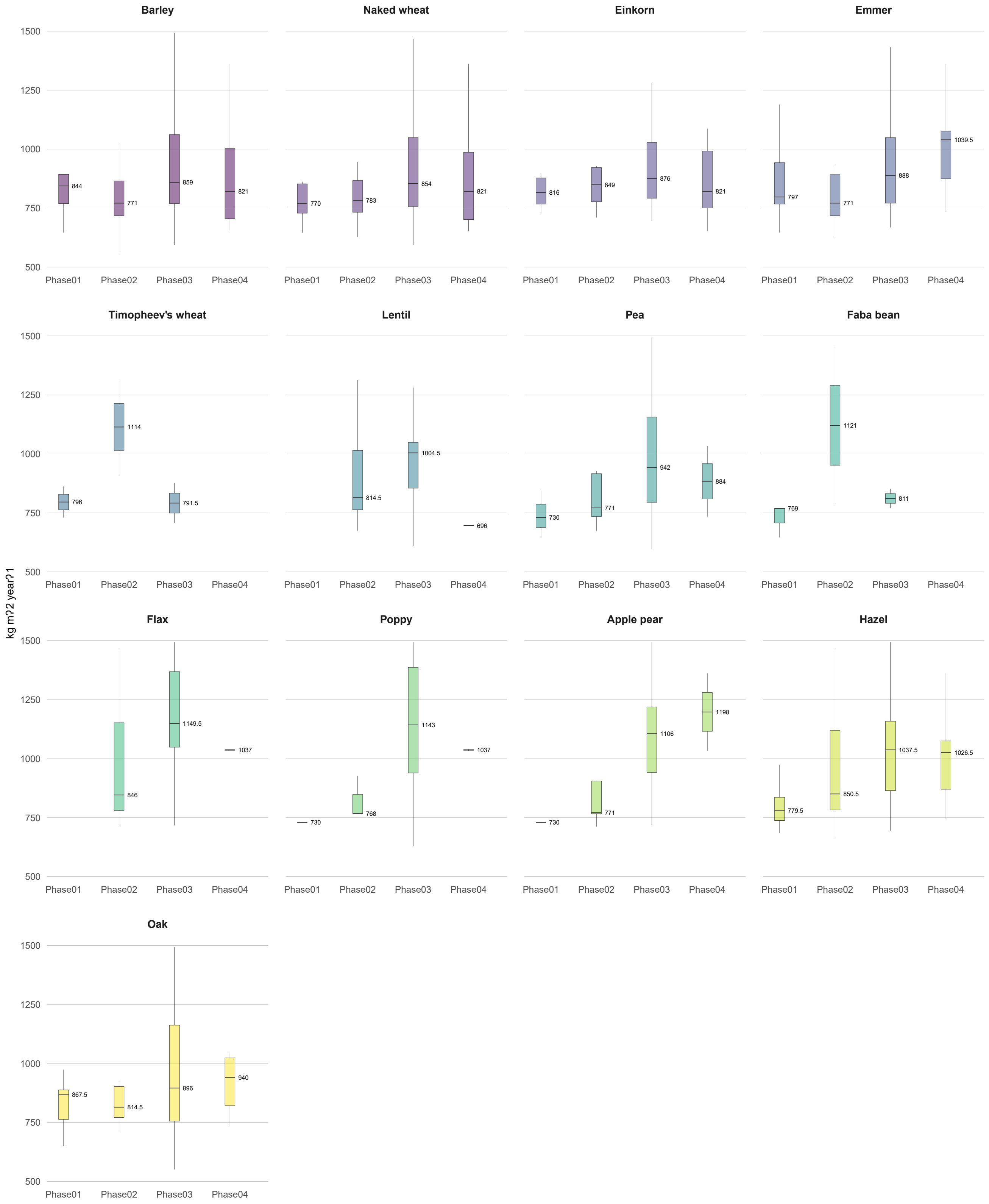

Supplementary Figure 8k: Distribution of crop occurrences over Annual Precipitation (Bio12) per Phase. The boxplots show medians, first and third quartiles (hinges), minimum and maximum values no further than 1.5*IQR from the hinge where IQR is the inter-quartile range (whiskers). Details about sample numbers are provided in Supplementary Table 3. Source data can be found on https://doi.org/10.5281/zenodo.14253277.

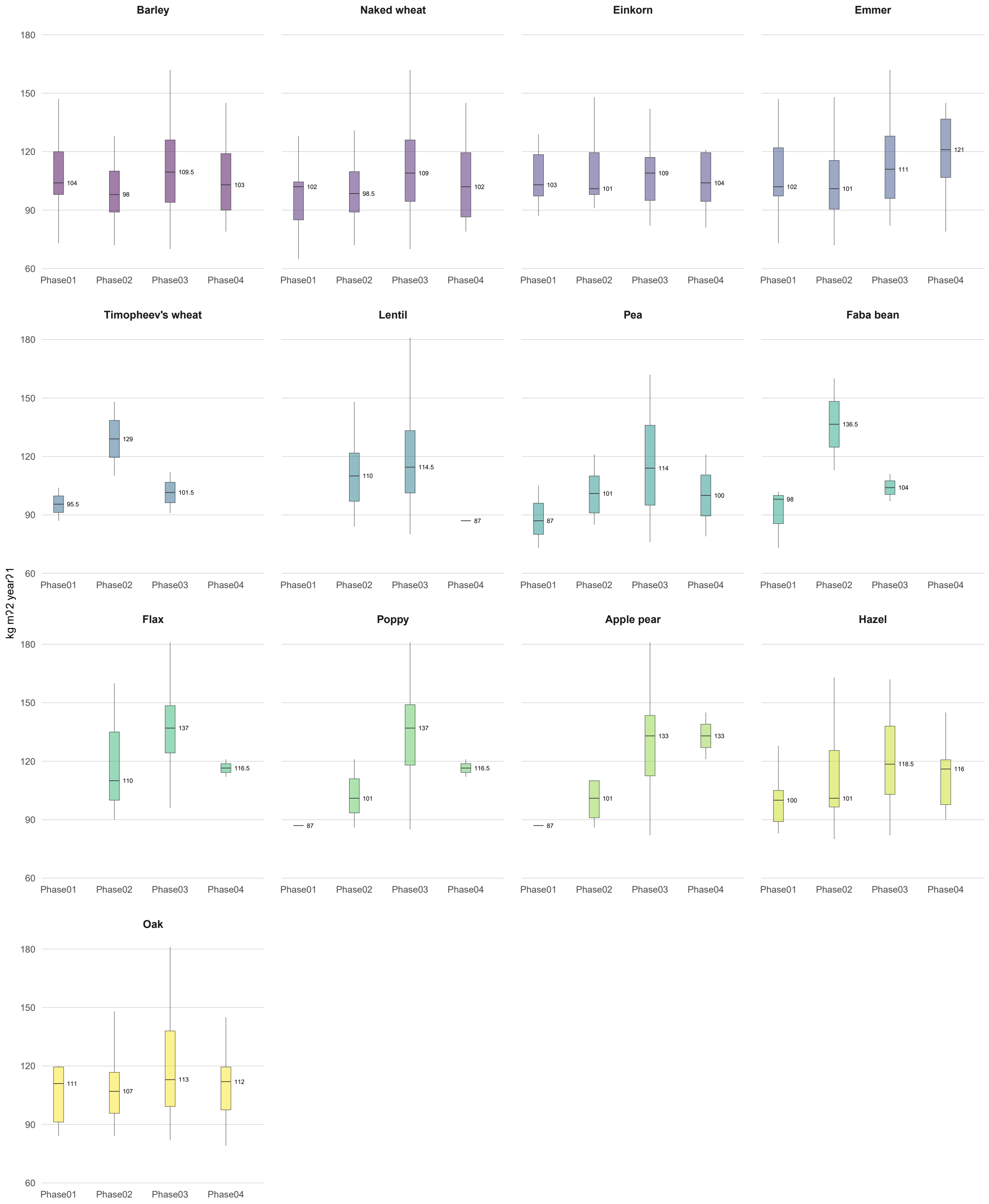

Supplementary Figure 8l: Distribution of crop occurrences over Precipitation of Wettest Month (Bio13) per Phase. The boxplots show medians, first and third quartiles (hinges), minimum and maximum values no further than 1.5*IQR from the hinge where IQR is the inter- quartile range (whiskers). Details about sample numbers are provided in Supplementary Table 3. Source data can be found on https://doi.org/10.5281/zenodo.14253277.

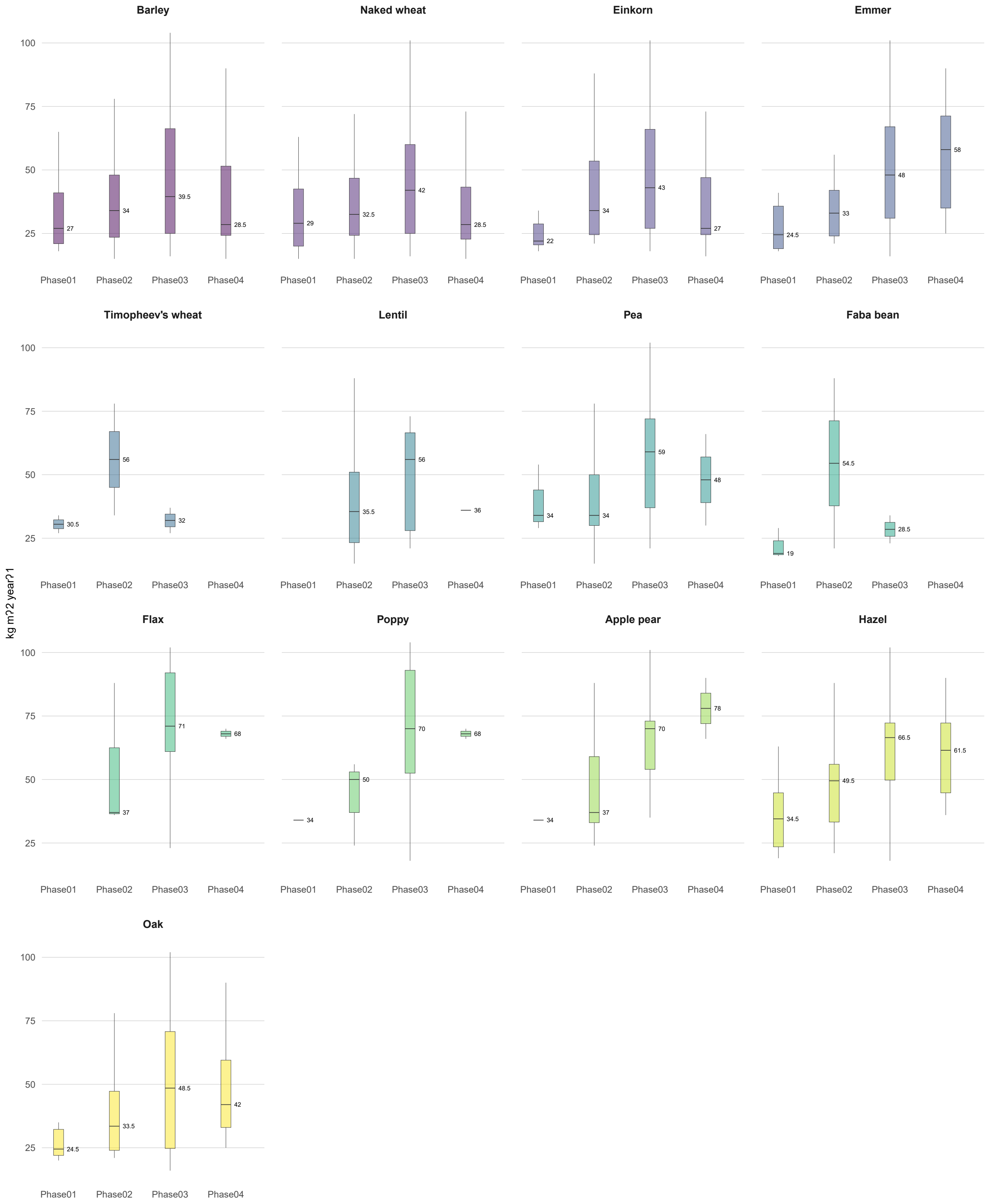

Supplementary Figure 8m: Distribution of crop occurrences over Precipitation of Driest Month (Bio14) per Phase. The boxplots show medians, first and third quartiles (hinges), minimum and maximum values no further than 1.5*IQR from the hinge where IQR is the inter- quartile range (whiskers). Details about sample numbers are provided in Supplementary Table 3. Source data can be found on https://doi.org/10.5281/zenodo.14253277.

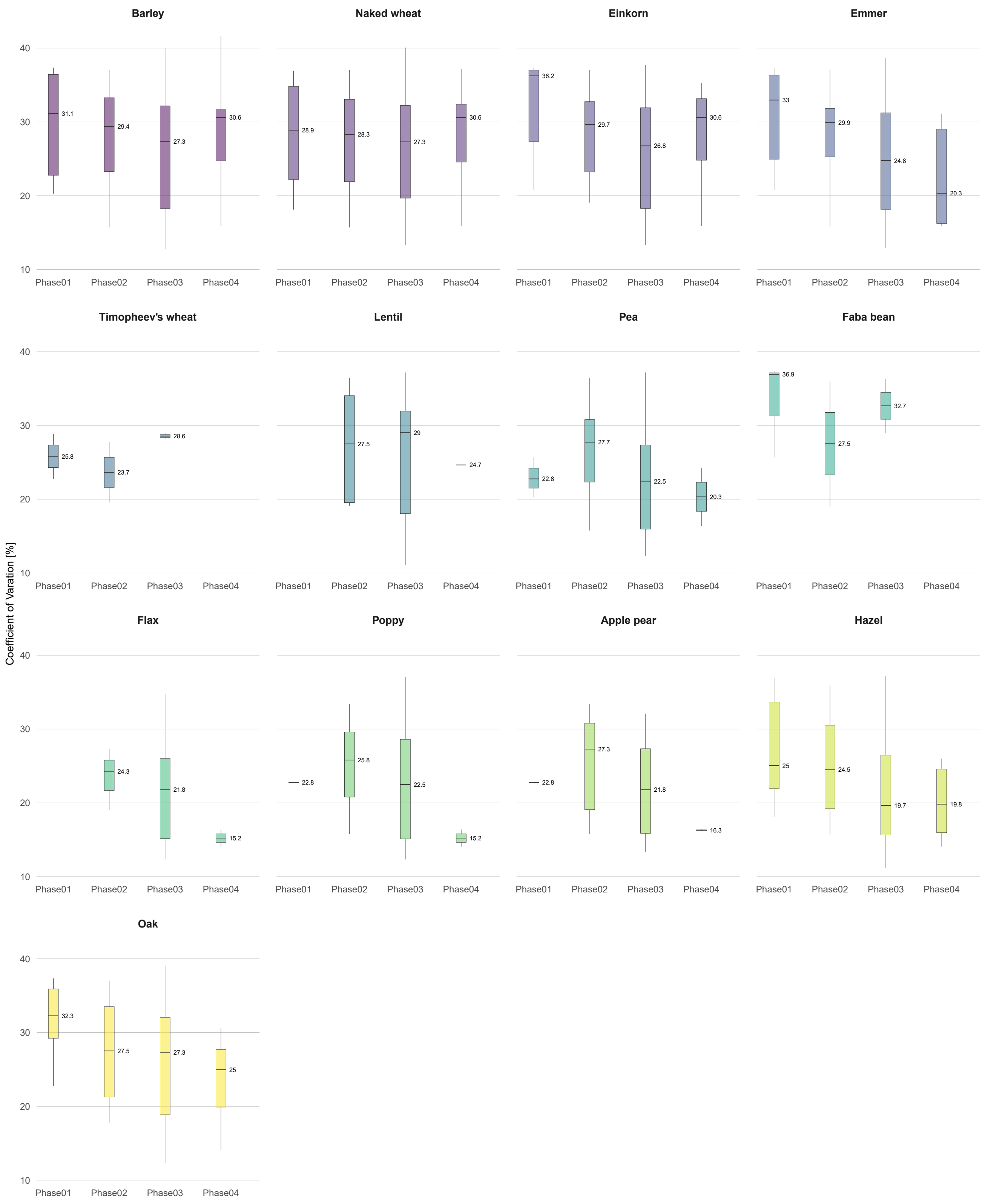

Supplementary Figure 8n: Distribution of crop occurrences over Precipitation Seasonality (Bio15) per Phase. The boxplots show medians, first and third quartiles (hinges), minimum and maximum values no further than 1.5*IQR from the hinge where IQR is the inter-quartile range (whiskers). Details about sample numbers are provided in Supplementary Table 3. Source data can be found on https://doi.org/10.5281/zenodo.14253277.

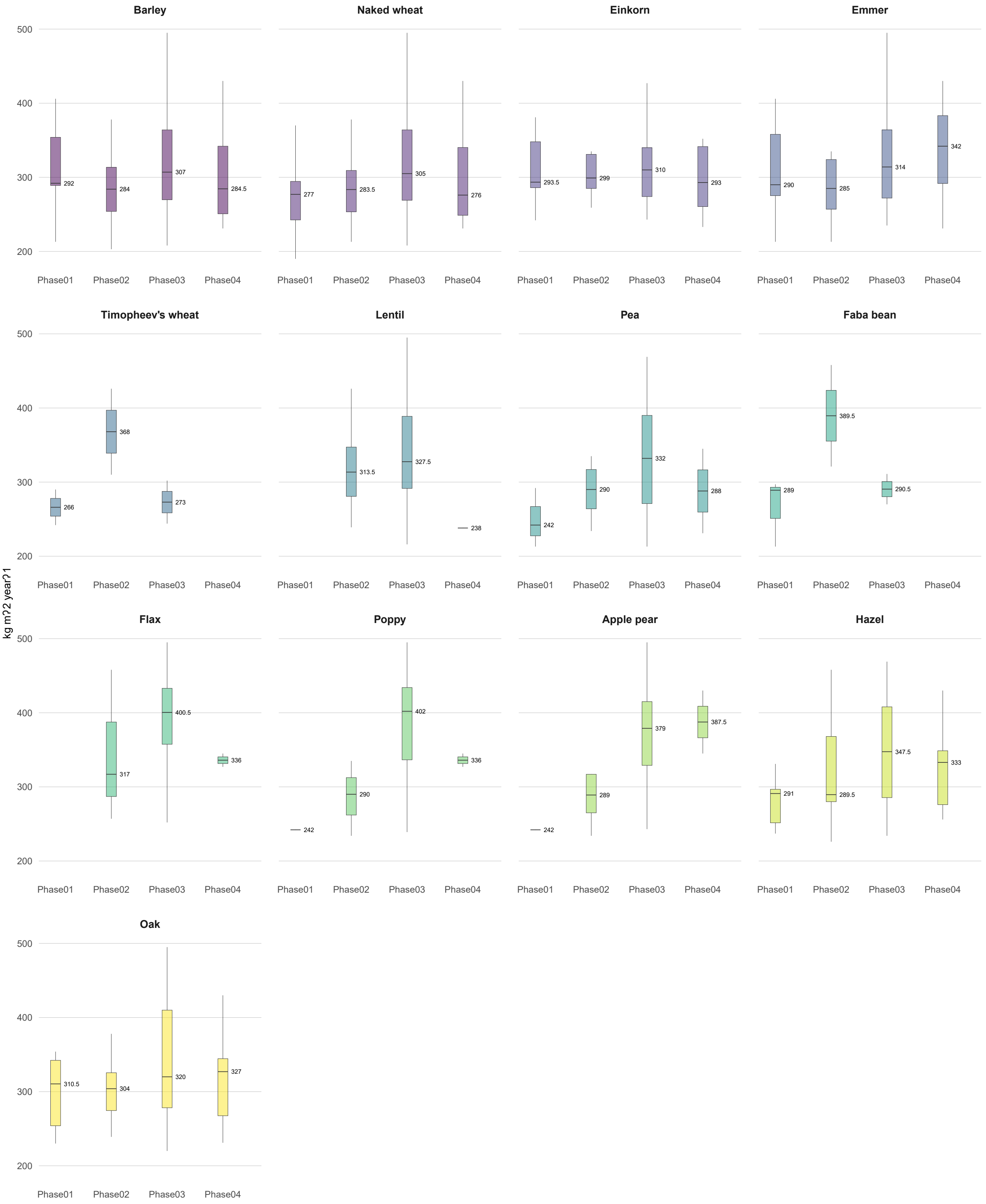

Supplementary Figure 8o: Distribution of crop occurrences over Precipitation of Wettest Quarter (Bio16) per Phase. The boxplots show medians, first and third quartiles (hinges), minimum and maximum values no further than 1.5*IQR from the hinge where IQR is the inter- quartile range (whiskers). Details about sample numbers are provided in Supplementary Table 3. Source data can be found on https://doi.org/10.5281/zenodo.14253277.

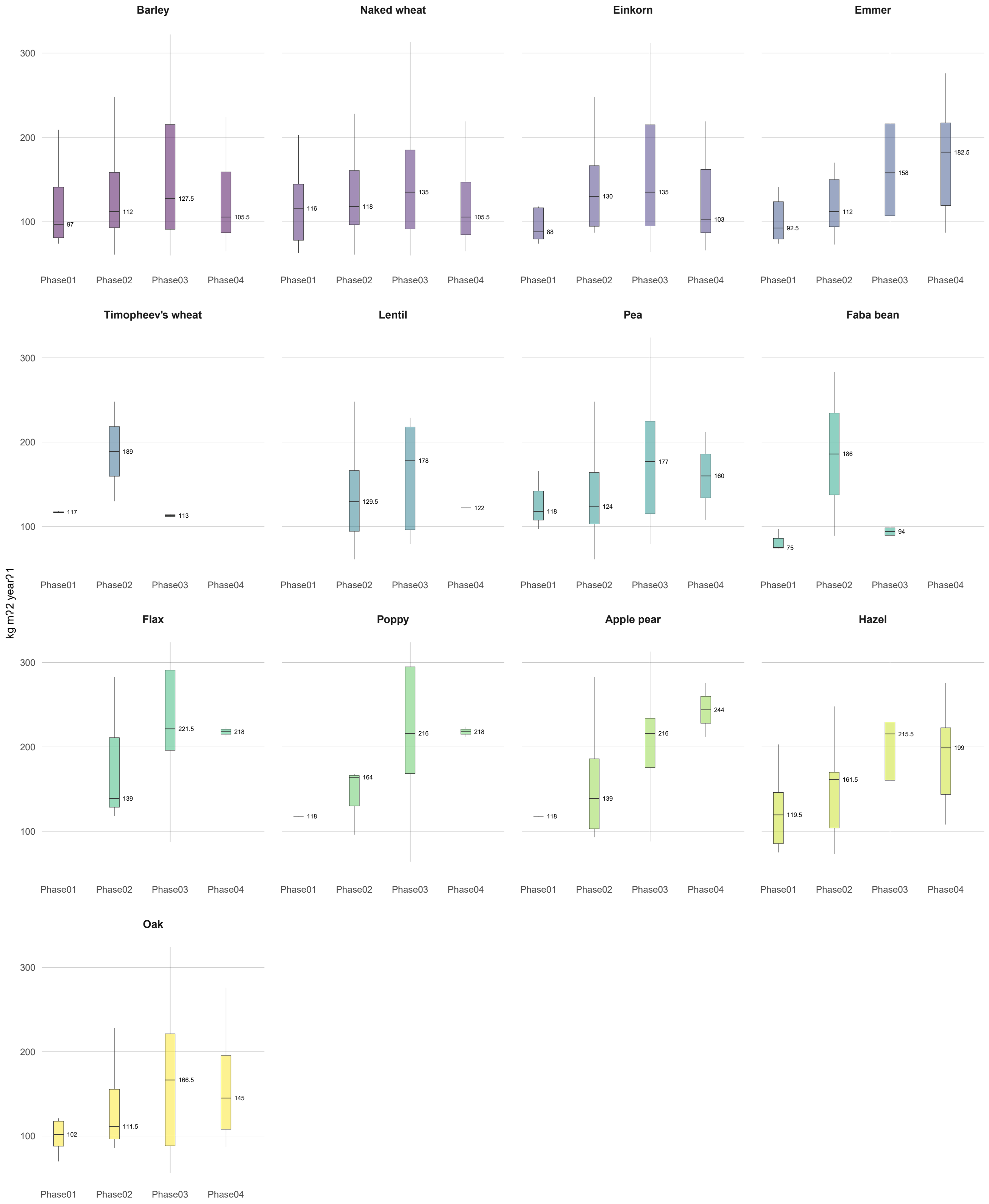

Supplementary Figure 8p: Distribution of crop occurrences over Precipitation of Driest Quarter (Bio17) per Phase. The boxplots show medians, first and third quartiles (hinges), minimum and maximum values no further than 1.5*IQR from the hinge where IQR is the inter- quartile range (whiskers). Details about sample numbers are provided in Supplementary Table 3. Source data can be found on https://doi.org/10.5281/zenodo.14253277.

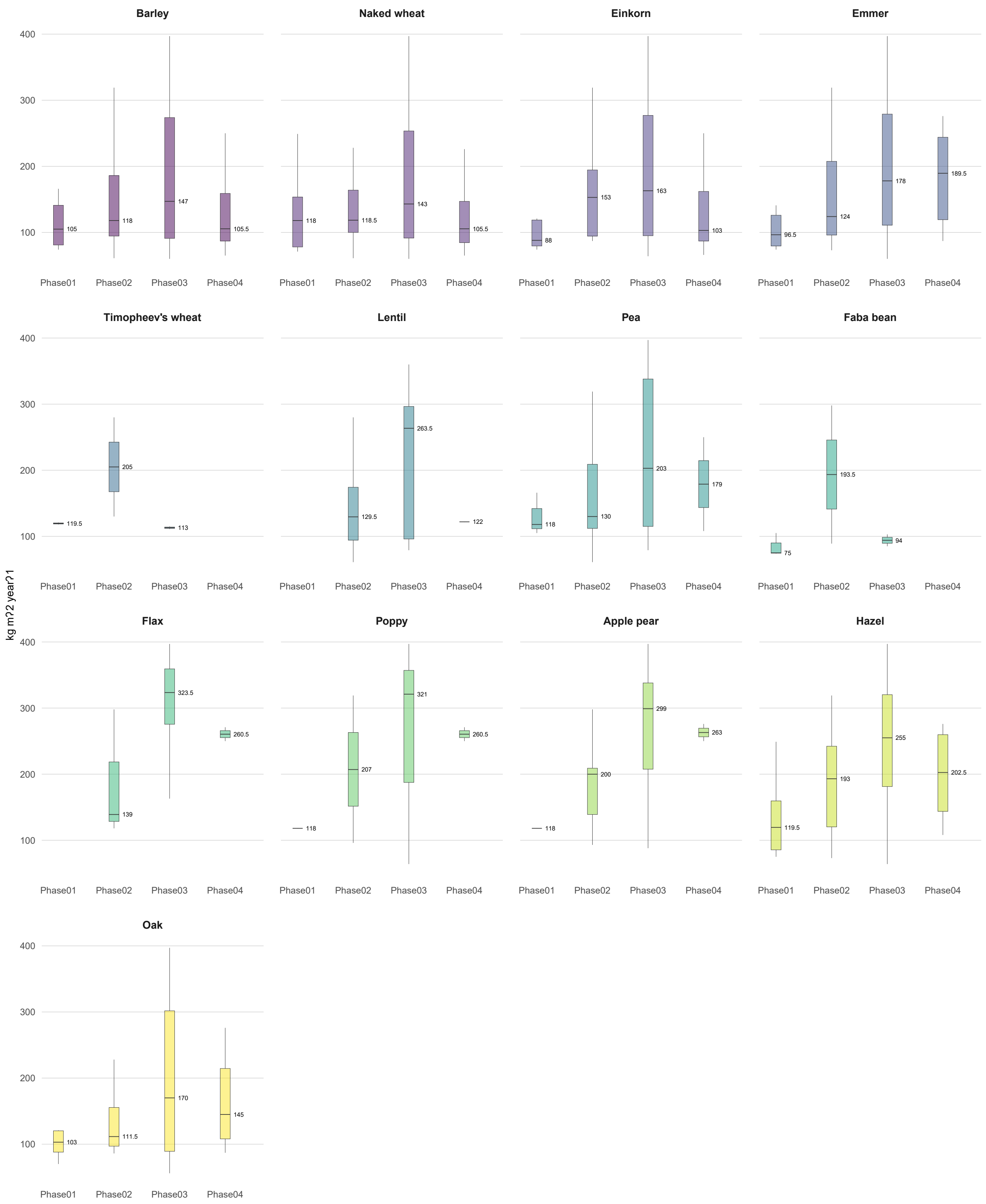

Supplementary Figure 8q: Distribution of crop occurrences over Precipitation of Warmest Quarter (Bio18) per Phase. The boxplots show medians, first and third quartiles (hinges), minimum and maximum values no further than 1.5*IQR from the hinge where IQR is the inter- quartile range (whiskers). Details about sample numbers are provided in Supplementary Table 3. Source data can be found on https://doi.org/10.5281/zenodo.14253277.

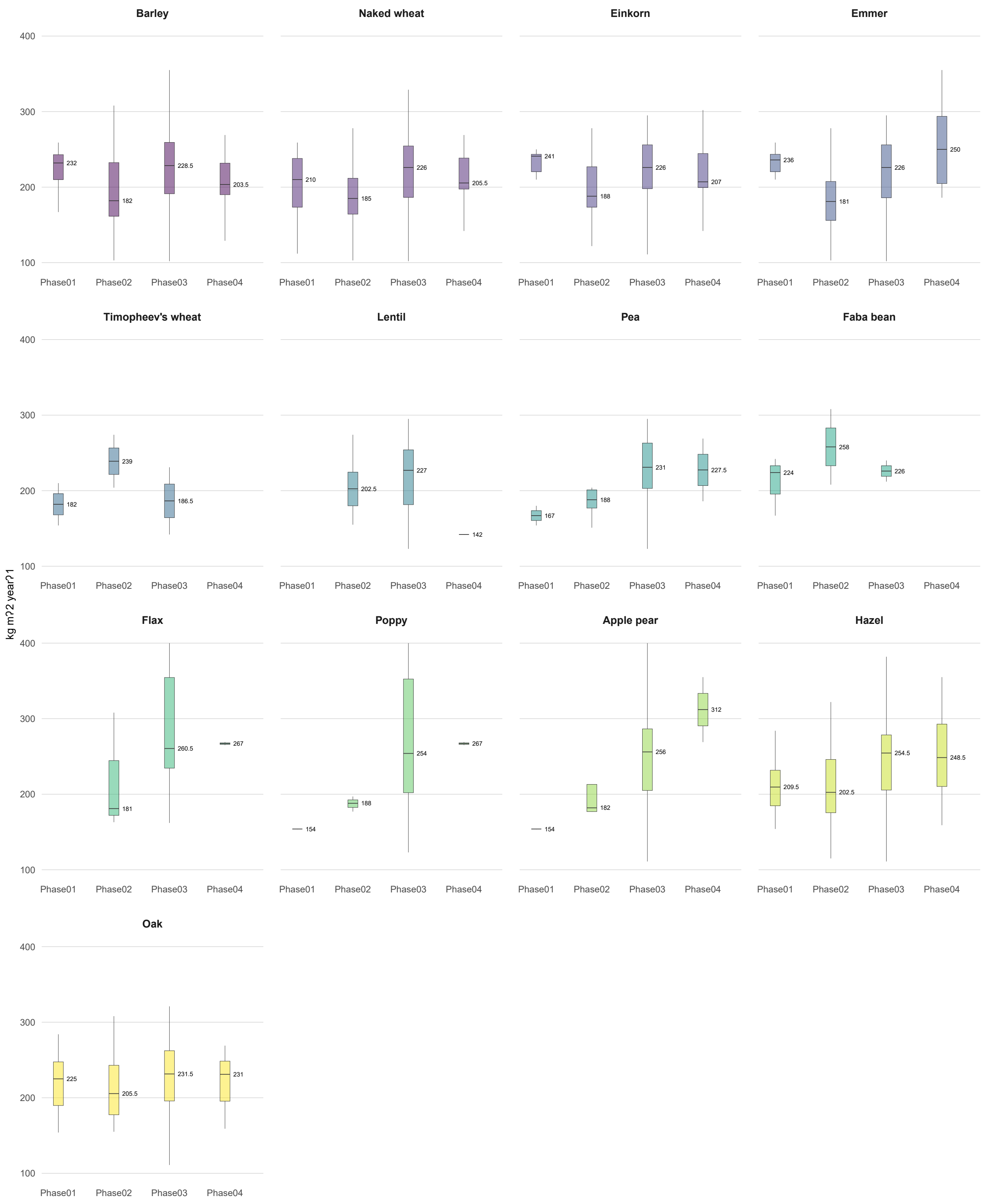

Supplementary Figure 8r: Distribution of crop occurrences over Precipitation of Coldest Quarter (Bio19) per Phase. The boxplots show medians, first and third quartiles (hinges), minimum and maximum values no further than 1.5*IQR from the hinge where IQR is the inter- quartile range (whiskers). Details about sample numbers are provided in Supplementary Table 3. Source data can be found on https://doi.org/10.5281/zenodo.14253277.

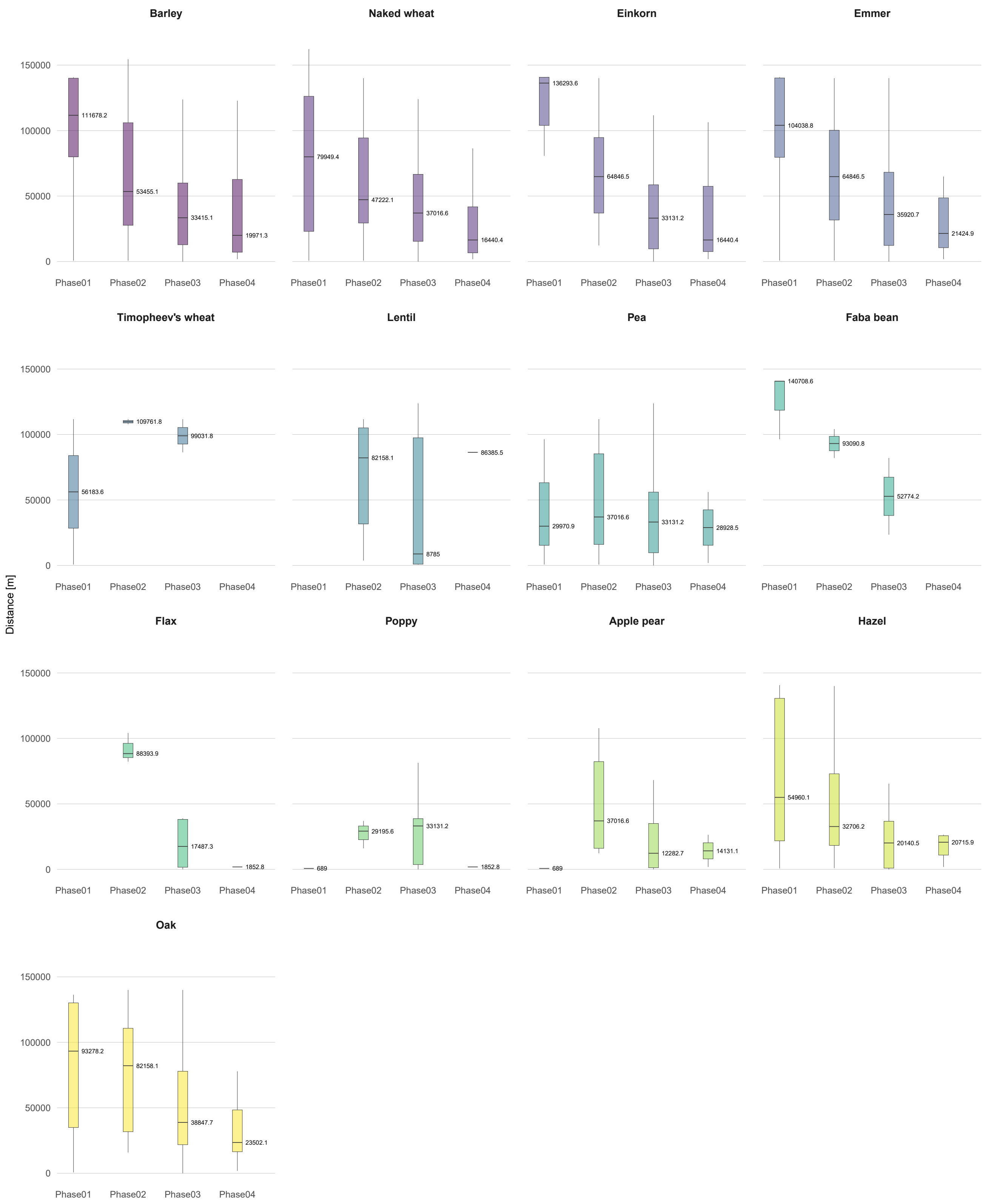

Supplementary Figure 8s: Distribution of crop occurrences over Distance to Lakes per Phase. The boxplots show medians, first and third quartiles (hinges), minimum and maximum values no further than 1.5*IQR from the hinge where IQR is the inter- quartile range (whiskers). Details about sample numbers are provided in Supplementary Table 3. Source data can be found on https://doi.org/10.5281/zenodo.14253277.

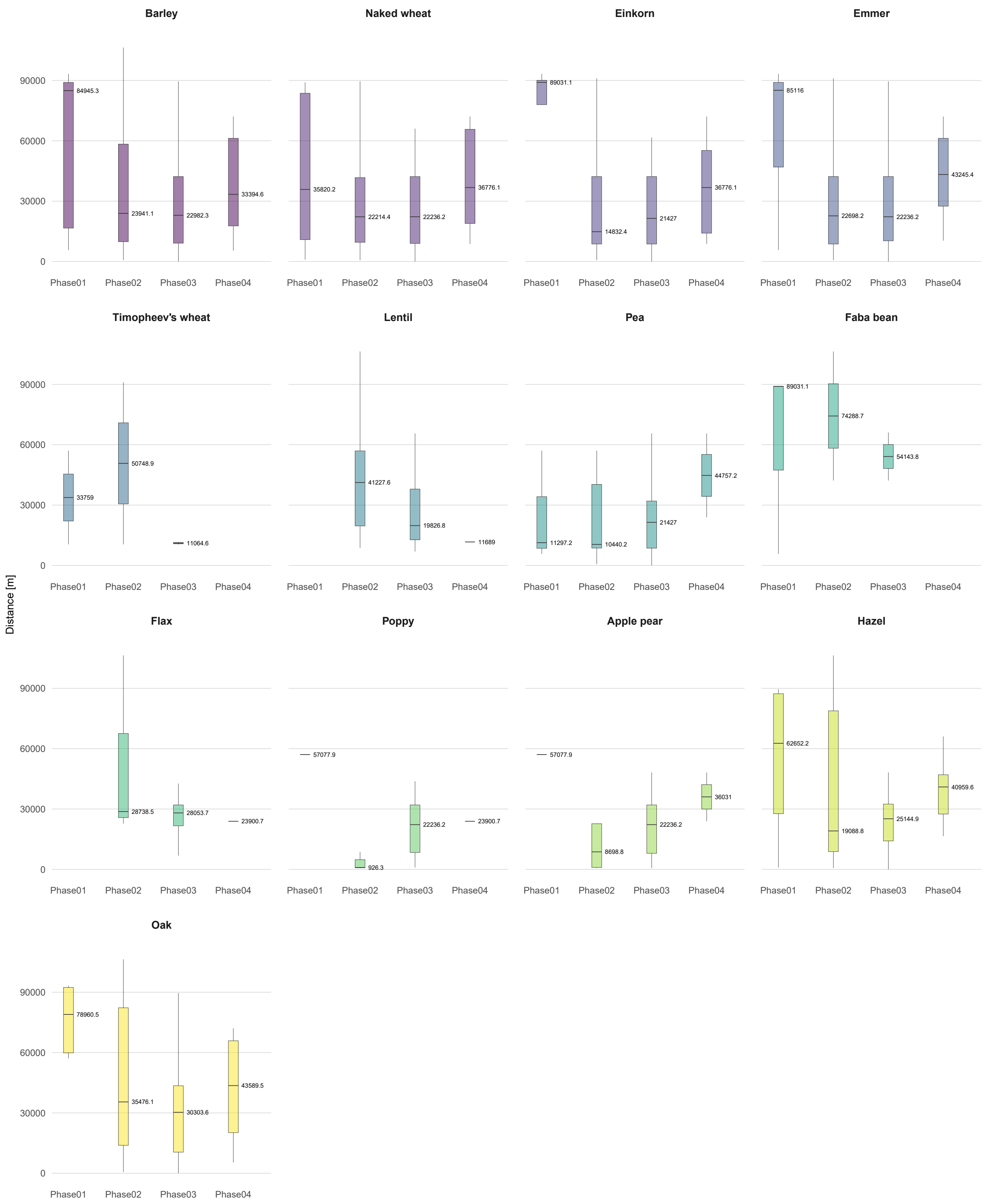

Supplementary Figure 8t: Distribution of crop occurrences over Distance to Rivers per Phase. The boxplots show medians, first and third quartiles (hinges), minimum and maximum values no further than 1.5*IQR from the hinge where IQR is the inter- quartile range (whiskers). Details about sample numbers are provided in Supplementary Table 3. Source data can be found on https://doi.org/10.5281/zenodo.14253277.

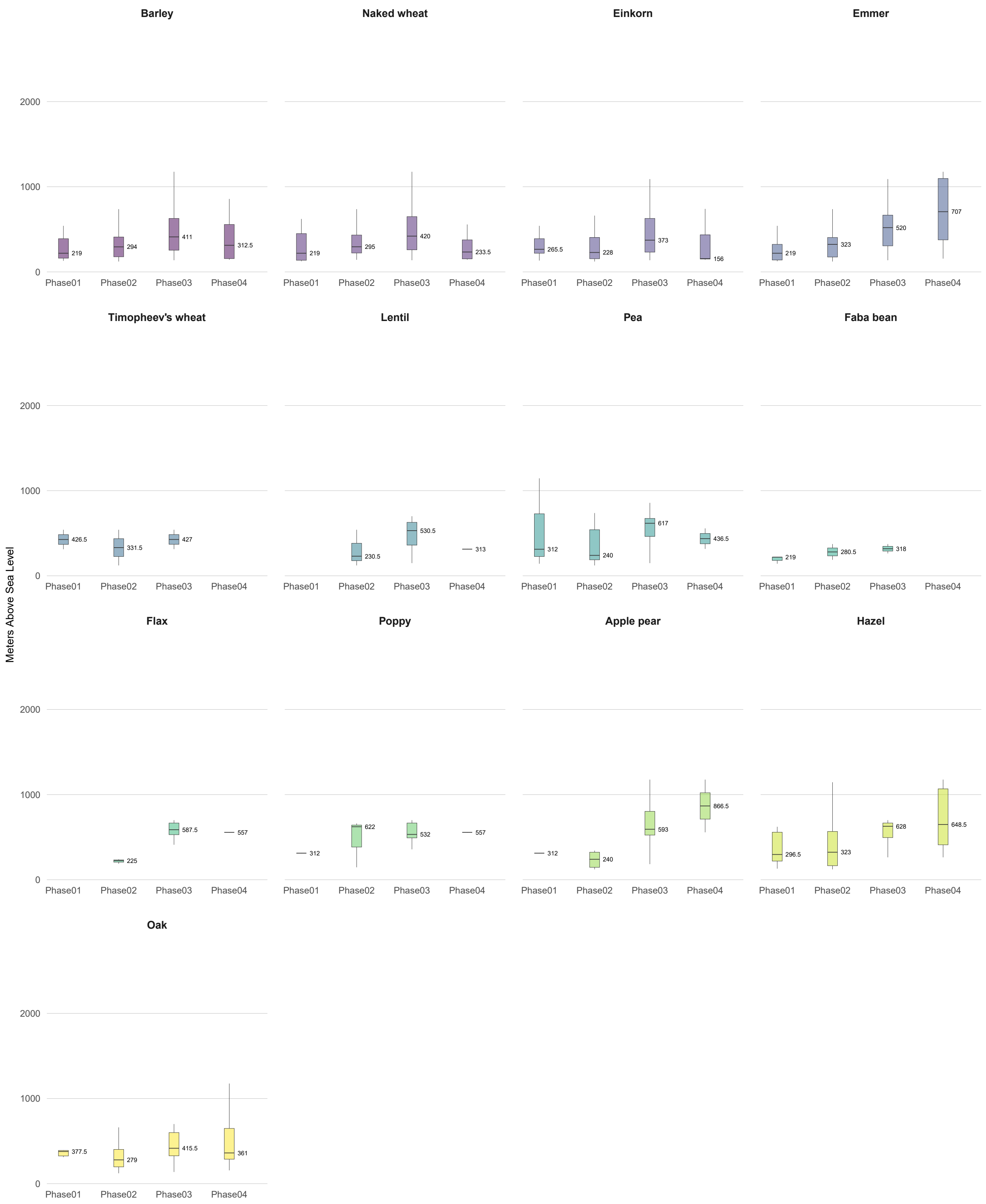

Supplementary Figure 8u: Distribution of crop occurrences over Altitude (DEM) per Phase. The boxplots show medians, first and third quartiles (hinges), minimum and maximum values no further than 1.5*IQR from the hinge where IQR is the inter- quartile range (whiskers). Details about sample numbers are provided in Supplementary Table 3. Source data can be found on https://doi.org/10.5281/zenodo.14253277.

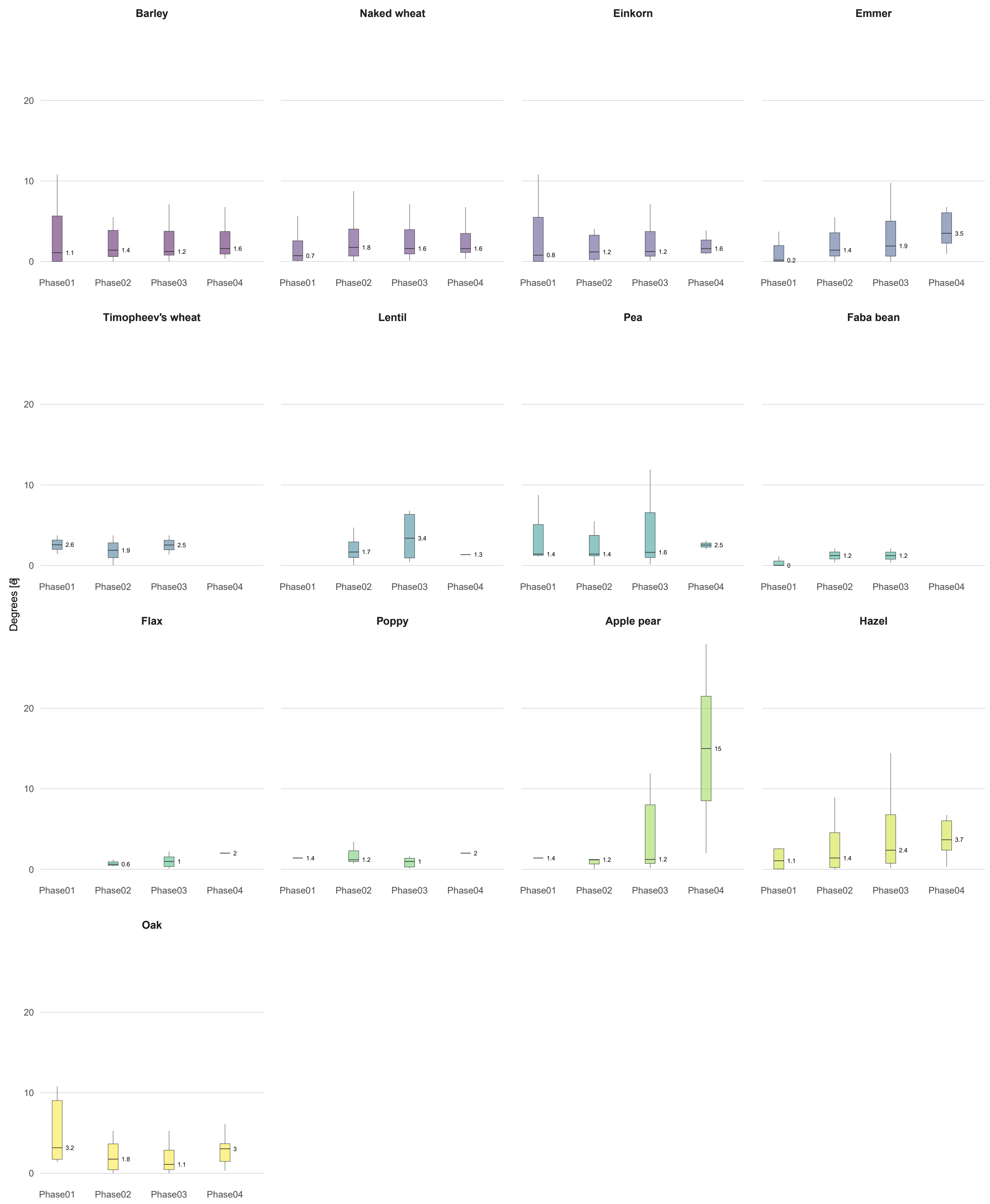

Supplementary Figure 8v: Distribution of crop occurrences over Slope per Phase. The boxplots show medians, first and third quartiles (hinges), minimum and maximum values no further than 1.5*IQR from the hinge where IQR is the inter- quartile range (whiskers). Details about sample numbers are provided in Supplementary Table 3. Source data can be found on https://doi.org/10.5281/zenodo.14253277.

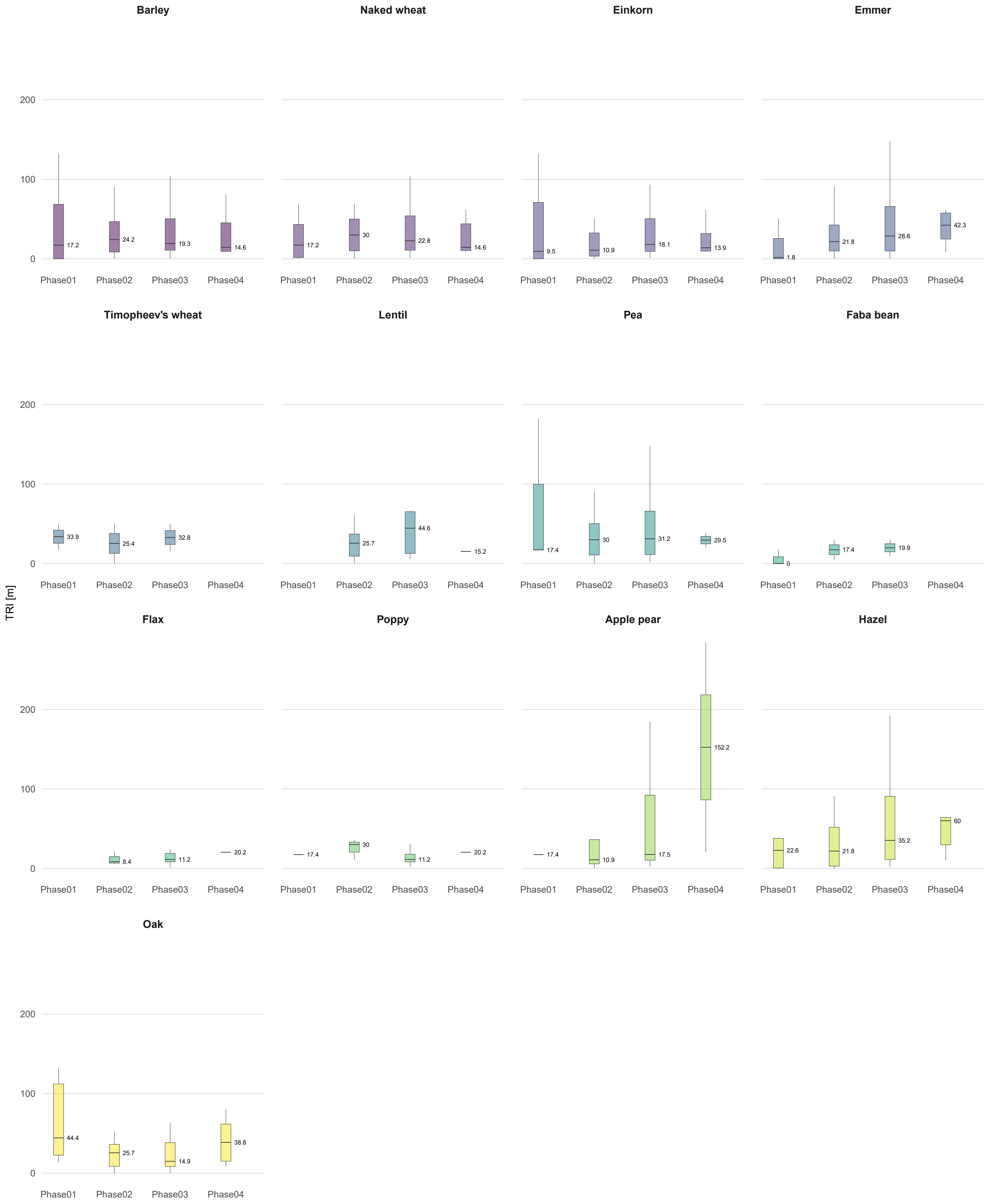

Supplementary Figure 8w: Distribution of crop occurrences over Terrain Ruggedness Index per Phase. The boxplots show medians, first and third quartiles (hinges), minimum and maximum values no further than 1.5*IQR from the hinge where IQR is the inter- quartile range (whiskers). Details about sample numbers are provided in Supplementary Table 3. Source data can be found on https://doi.org/10.5281/zenodo.14253277.

Crop	Phase 01	Phase 02	Phase 03	Phase 04	Total	Percentage
Barley	13	40	92	22	167	19.8 %
Naked Wheat	11	34	75	16	136	16.1 %
Einkorn	8	15	49	11	83	9.8 %
Emmer	12	27	57	6	102	12.1 %
Thimopheev's wheat	2	2	2	0	6	0.7 %
Lentil	0	8	10	1	19	2.3 %
Pea	3	13	29	2	47	5.6 %
Fava Bean	3	2	2	0	7	0.8 %
Flax	0	3	24	2	29	3.4 %
Poppy	1	3	27	2	33	3.9 %
Apple pear	1	9	27	2	39	4.6 %
Hazel	10	26	48	6	90	10.7 %
Oak	6	18	50	11	85	10.1 %
Total	70	200	492	81	843	100 %

Supplementary Table 3: Crop types and numbers. The table shows the total number and relative frequency in percentage of each crop type.

Label	Name	Unit	Description
Paleo-climatic variables			
Bio01	Annual Mean Temperature	°C	Mean of monthly (maximum near-surface (2 m) air temperature + minimum near-surface (2 m) air temperature) / 2
Bio02	Mean Diurnal Range	°C	The annual mean of all the monthly diurnal temperature ranges. Each monthly diurnal range is the difference between that month's maximum and minimum temperature.
Bio04	Temperature Seasonality	°C * 100	The standard deviation of the monthly mean temperatures.
Bio05	Maximum Temperature of Warmest Month	°C	The highest temperature of any monthly maximum temperature.
Bio06	Minimum Temperature of Coldest Month	°C	The lowest temperature of any monthly minimum temperature.
Bio07	Temperature Annual Range	°C	The difference between the Maximum Temperature of Warmest Period and the Minimum Temperature of Coldest Period.
Bio08	Mean Temperature of Wettest Quarter	°C	The wettest quarter of the year is determined (to the nearest month), and the mean temperature of this period is calculated.
Bio09	Mean Temperature of Driest Quarter	°C	The driest quarter of the year is determined (to the nearest month), and the mean temperature of this period is calculated.
Bio10	Mean Temperature of Warmest Quarter	°C	The warmest quarter of the year is determined (to the nearest month), and the mean temperature of this period is calculated.
Bio11	Mean Temperature of Coldest Quarter	°C	The coldest quarter of the year is determined (to the nearest month), and the mean temperature of this period is calculated.
Bio12	Annual Precipitation	kg m-2 year-1 (mm / year)	The sum of all the monthly precipitation estimates.
Bio13	Precipitation of Wettest Month	kg m-2 year-1 (mm / year)	The precipitation of the wettest month.
Bio14	Precipitation of Driest Month	kg m-2 year-1 (mm / year)	The precipitation of the driest month.
Bio15	Precipitation Seasonality	unitless	The Coefficient of Variation is the standard deviation of the monthly precipitation estimates expressed as a percentage of the mean of those estimates (i.e. the annual mean).
Bio16	Precipitation of Wettest Quarter	kg m-2 year-1 (mm / year)	The wettest quarter of the year is determined (to the nearest month), and the total precipitation over this period is calculated.
Bio17	Precipitation of Driest Quarter	kg m-2 year-1 (mm / year)	The driest quarter of the year is determined (to the nearest month), and the total precipitation over this period is calculated.
Bio18	Precipitation of Warmest Quarter	kg m-2 year-1 (mm / year)	The warmest quarter of the year is determined (to the nearest month), and the total precipitation over this period is calculated.
Bio19	Precipitation of Coldest Quarter	kg m-2 year-1 (mm / year)	The coldest quarter of the year is determined (to the nearest month), and the total precipitation over this period is calculated.
***Environmental variable*s**			
DEM	Digital Elevation Model	m.a.s.l.	Surface elevation above sea level.
Slope	Slope	Degrees [°]	Slope computed using terrain function from the *terra* package in *R*
TRI	Terrain Ruggedness Index	m	TRI computed using terrain function from the *terra* package in *R*
D_Lakes	Distance from main lakes	m	Geodesic Euclidean distance from reconstructed main lakes (from Martinez-Grau et al. 2021).
D_Rivers	Distance from main rivers	m	Geodesic Euclidean distance from reconstructed main rivers (from Martinez-Grau et al. 2021).

Supplementary Table 4: Set of variables paleoclimatic and environmental variables. Label, Name, Unit and Description of the paleoclimatic (adapted from Karger et al. 2020) and the environmental variables used for the modelling procedure.

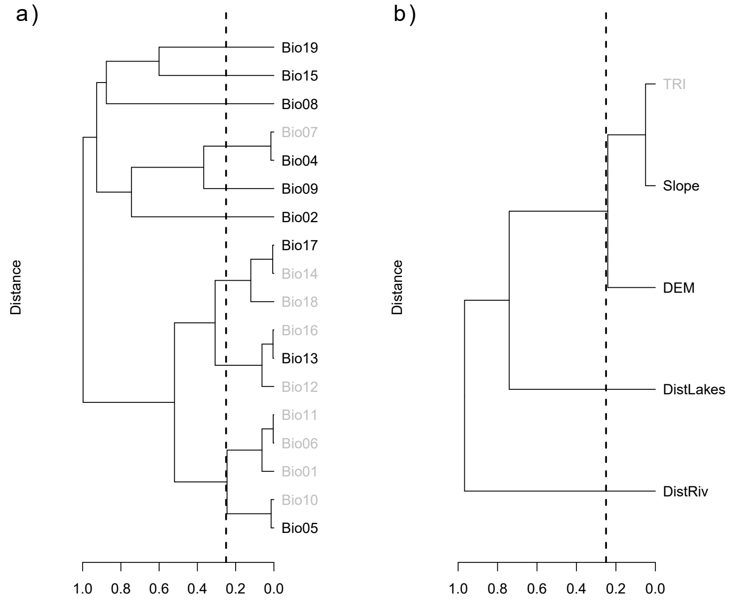

Supplementary Figure 9: Correlation dendrograms for paleoclimatic (a) and environmental (b) predictors. Correlation analysis using non-parametric Spearman correlation tests. The dashes line shows the correlation limit of r=0.25 applied for predictor selection, the highlighted variables represent the retained predictors. Bio01: Annual Mean Temperature; Bio02: Mean Diurnal Range; Bio04: Temperature Seasonality; Bio05: Maximum Temperature of Warmest Month; Bio06: Minimum Temperature of Coldest Month; Bio07: Temperature Annual Range; Bio08: Mean Temperature of Wettest Quarter; Bio09: Mean Temperature of Driest Quarter; Bio10: Mean Temperature of Warmest Quarter; Bio11: Mean Temperature of Coldest Quarter; Bio12: Annual Precipitation; Bio13: Precipitation of Wettest Month; Bio14: Precipitation of Driest Month; Bio15: Precipitation Seasonality; Bio16: Precipitation of Wettest Quarter; Bio17: Precipitation of Driest Quarter; Bio18: Precipitation of Warmest Quarter; Bio19: Precipitation of Coldest Quarter; DEM: Digital Elevation Model; Slope: Slope; TRI: Terrain Ruggedness Index; DistLakes: Distance from main lakes; DistRiv: Distance from main rivers. Source data can be found on https://doi.org/10.5281/zenodo.14253277.

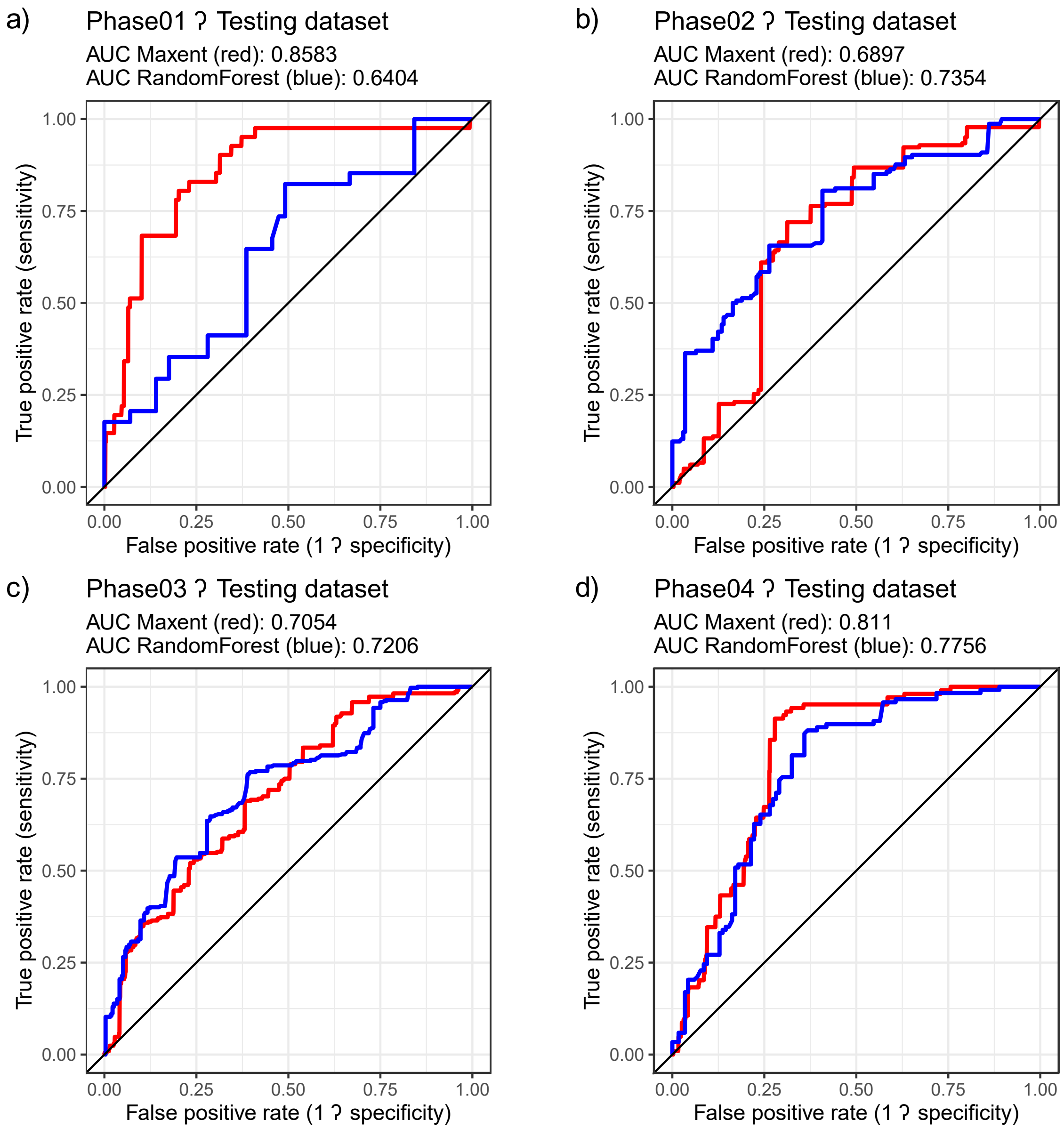

Supplementary Figure 10: AUC-ROC. Receiver Operating Characteristic (ROC curves) and Area Under the Curve (AUC) for Testing Datasets using Random Forest (blue line) and MaxEnt (red line) for Phase 01 (a), Phase 02 (b), Phase 03 (c) and Phase 04 (d). Source data can be found on https://doi.org/10.5281/zenodo.14253277.

Supplementary Reference

Karger, Dirk Nikolaus; Nobis, Michael P.; Normand, Signe; Graham, Catherine H.; Zimmermann, Niklaus E. (2020). CHELSA-TraCE21k: Downscaled transient temperature and precipitation data since the last glacial maximum. EnviDat. https://doi.org/10.16904/envidat.211
